# Knowledge-based matrix factorization temporally resolves the cellular responses to IL-6 stimulation

**DOI:** 10.1186/1471-2105-11-585

**Published:** 2010-11-30

**Authors:** Andreas Kowarsch, Florian Blöchl, Sebastian Bohl, Maria Saile, Norbert Gretz, Ursula Klingmüller, Fabian J Theis

**Affiliations:** 1 Institute for Bioinformatics and Systems Biology, Helmholtz Zentrum München, Neuherberg, Germany; 2 Division of Systems Biology of Signal Transduction, DKFZ-ZMBH Alliance, German Cancer Research Center, Heidelberg, Germany; 3 Medical Research Center, Medical Faculty Mannheim, University Heidelberg, Mannheim, Germany; 4 Department of Mathematical Science, Technische Universität München, Garching, Germany

## Abstract

**Background:**

External stimulations of cells by hormones, cytokines or growth factors activate signal transduction pathways that subsequently induce a re-arrangement of cellular gene expression. The analysis of such changes is complicated, as they consist of multi-layered temporal responses. While classical analyses based on clustering or gene set enrichment only partly reveal this information, matrix factorization techniques are well suited for a detailed temporal analysis. In signal processing, factorization techniques incorporating data properties like spatial and temporal correlation structure have shown to be robust and computationally efficient. However, such correlation-based methods have so far not be applied in bioinformatics, because large scale biological data rarely imply a natural order that allows the definition of a delayed correlation function.

**Results:**

We therefore develop the concept of graph-decorrelation. We encode prior knowledge like transcriptional regulation, protein interactions or metabolic pathways in a weighted directed graph. By linking features along this underlying graph, we introduce a partial ordering of the features (e.g. genes) and are thus able to define a graph-delayed correlation function. Using this framework as constraint to the matrix factorization task allows us to set up the fast and robust graph-decorrelation algorithm (GraDe). To analyze alterations in the gene response in *IL-6 *stimulated primary mouse hepatocytes, we performed a time-course microarray experiment and applied GraDe. In contrast to standard techniques, the extracted time-resolved gene expression profiles showed that *IL-6 *activates genes involved in cell cycle progression and cell division. Genes linked to metabolic and apoptotic processes are down-regulated indicating that *IL-6 *mediated priming renders hepatocytes more responsive towards cell proliferation and reduces expenditures for the energy metabolism.

**Conclusions:**

GraDe provides a novel framework for the decomposition of large-scale 'omics' data. We were able to show that including prior knowledge into the separation task leads to a much more structured and detailed separation of the time-dependent responses upon *IL-6 *stimulation compared to standard methods. A Matlab implementation of the GraDe algorithm is freely available at http://cmb.helmholtz-muenchen.de/grade.

## Background

With the availability of high-throughput 'omics' data, more and more methods from statistics and signal processing are applied in the field of bioinformatics [1]. Direct application of such methods to biological data sets is essentially complicated by three issues, namely *(i) *the large-dimensionality of observed variables (e.g. transcripts or metabolites), *(ii) *the small number of independent experiments and *(iii) *the necessity to take into account prior information in the form of e.g. interaction networks or chemical reactions. While *(i) *may be tackled by targeted analysis, feature selection or efficient dimension reduction methods, the issue of low number of samples (experiments) may hinder the transfer of methods. For example, with cDNA microarrays, the number of genes (*p*) is usually much larger than the experiment size *n *(number of arrays). Quantitative data from experiments are often classified as 'small-*n*-large-*p*' problems [2] and algorithms that are currently being developed are tailored for such kind of data. Detailed prior information is in general best handled by Bayesian methods [3], which are however not straight-forward to formulate in small-*n*-large-*p *problems.

Here, we focus on the unsupervised extraction of overlapping clusters in data sets exhibiting properties *(i-iii)*. If applied to gene expression profiles acquired by microarrays or metabolic profiles from mass spectrometry, we can interpret these clusters as jointly acting species (cellular processes). While partitioned clustering based on *k*-means [4] or hierarchical clustering [5] has been successful in some domains and is often the initial tool of choice for data grouping, overlapping clusters are better described by fuzzy techniques [6] or linear models [7]. Focusing on the latter, we can essentially summarize these techniques as matrix factorization algorithms. Constraining the factorization using e.g. decorrelation, statistical independence or non-negativity then leads to algorithms like principal component analysis (PCA), independent component analysis [8] and nonnegative matrix factorization [9], respectively. Although such methods are successfully applied in bioinformatics [10-12], they partially run into issues *(i-iii) *as described above. In particular, it is not clear how to include prior knowledge, which has been a quite successful strategy in other contexts [13]. A first step towards this direction is network component analysis (NCA) [14,15]. It integrates prior knowledge in form of a multiple-input motif to uncover hidden regulatory signals from the outputs of networked systems, a task also covered in [16]. Hence, it focuses on the estimation of single gene's expression profiles, not in a linear decomposition of a data set into overlapping clusters. NCA poses strict assumptions on the topology of this predefined network, which makes it hardly applicable to mammalian high-throughput 'omics' data. Moreover, feedbacks from the regulated species back to the regulators are treated only as 'closed-loops', without explicitly modeling the feedback structure.

To overcome these constraints, this contribution provides a novel framework for the linear decomposition of data sets into expression profiles. We present a new matrix factorization method that is computationally efficient *(i)*, able to deal with the low number of experiments *(ii) *and includes as much prior information as possible *(iii)*. In order to achieve computational efficiency coupled with robust estimation, we use delayed correlations instead of higher-order statistics. In signal processing, this strategy has been shown to be advantageous [17,18] for two reasons: such methods use more information from the data without over-fitting it and they can be formulated as second order techniques. This is crucial for the application to microarray data, since dimensionality tends to be high in this environment.

However, delayed correlations can usually not be computed in the case of biological high-throughput experiments such as in microarray samples. While time-resolved experiments may provide correlations, the number of temporal observations is commonly too small (<10) for the estimation of time-delayed correlations.

**Figure 1:**
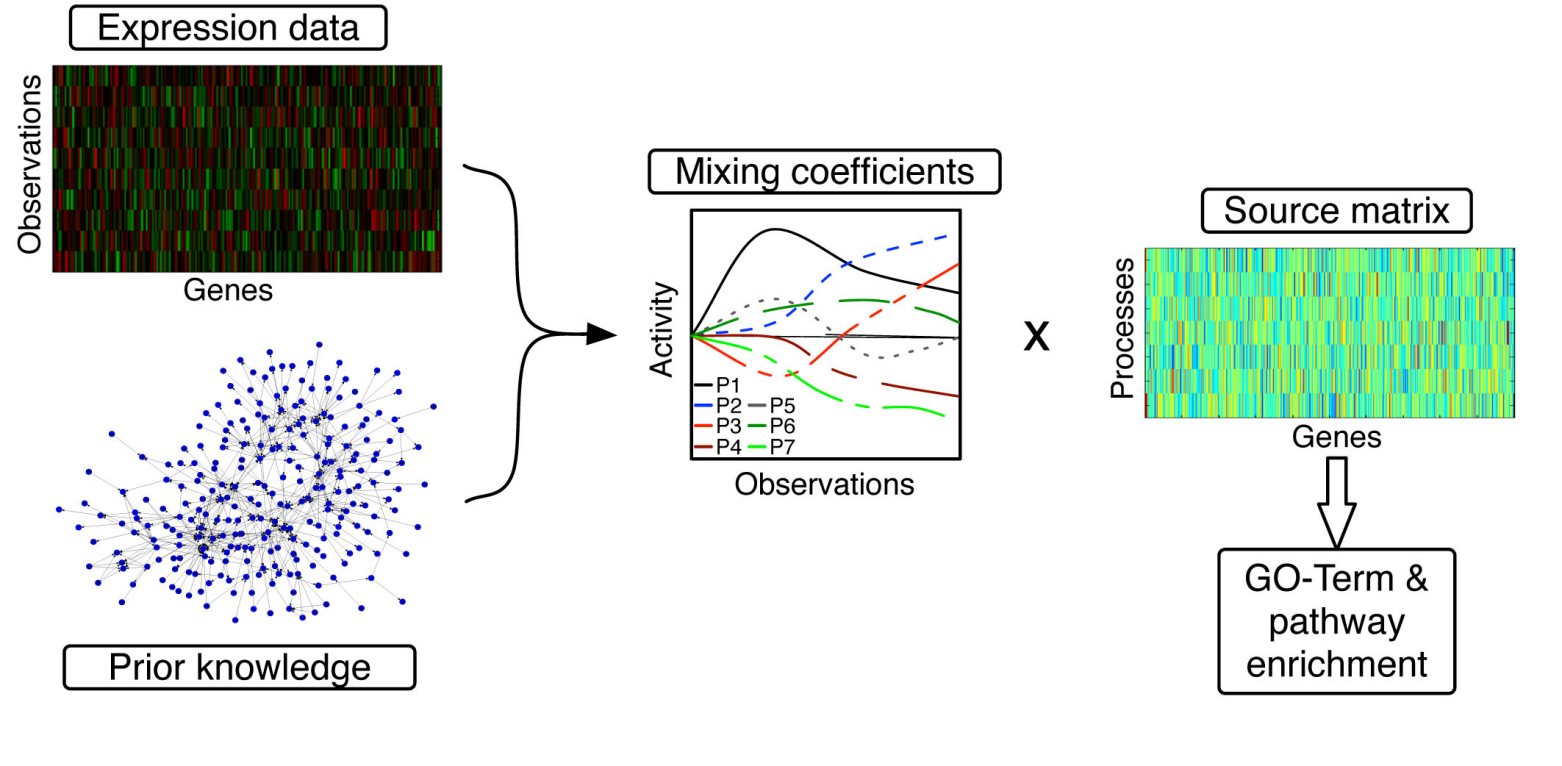
**GraDe: Graph-decorrelation algorithm**. In cells, various biological processes are taking place simultaneously. Each of these processes has its own characteristic gene expression pattern, but different processes may overlap. A cell's total gene expression is then the sum of the expression patterns of all active processes, weighted by their current activation level. The GraDe algorithm combines a matrix factorization approach with prior knowledge in form of an underlying regulatory network. The input of GraDe is the transcriptional expression data, where observations can be different conditions or a time points, and the underlying regulatory network (prior knowledge). GraDe decomposes the observed expression data into the underlying sources *S *and their mixing coefficients A. Analyzing time-course microarray data, we interpret these sources as the biological processes and the mixing coefficients as their time-dependent activities. Observations indicate their expression behavior either in the different conditions or time-points and activity their activation strength. We further filter process-related genes by taking only the genes with the strongest contribution in each process. Finally, we test for enrichment of cellular processes (GO) and biological pathways (KEGG).

Hence, we instead pose factorization conditions along the set of genes or other biological variables. We link these variables using prior knowledge e.g. in the form of a transcription factor or protein-protein interaction (PPI) network, metabolic pathways or *via *explicitly given models. Using this information enables us to define a graph-decorrelation algorithm that combines prior knowledge with source-separation techniques, for illustration see Figure 1. In case of gene expression analysis the input of GraDe are the expression data and an underlying regulatory network. After applying GraDe, we obtain two matrices, a mixing and a source matrix. We interpret the sources as the biological processes and the mixing coefficients as their time-dependent activities. Hereby, the extracted sources group the genes' expression that can be explained by the underlying regulatory network, e.g. different responses of a cell to an external stimulus.

The cytokine interleukin IL-6 mediates the production of acute phase proteins by hepatocytes and promotes liver regeneration [19]. In order to unveil the multi-layered temporal gene responses in these processes, we measure gene expression in *IL-6 *stimulated mouse hepatocytes by a time-course microarray experiment. Applying GraDe with a literature based gene regulatory network, we are able to infer associated biological processes as well as the dynamic behavior of *IL-6 *related gene expression. In addition, we find that the estimated factors are robust against the high number of false positives contained in large-scale biological databases.

## Results and Discussion

The activation of gene regulatory processes upon external stimulations induces a re-arrangement of cellular gene expression patterns. Matrix factorization techniques are currently explored in the analysis of such multi-layered and overlapping temporal responses. In the following, we propose an algorithm that incorporates prior knowledge as a constraint to the factorization task (see Figure 1).

### Algorithm: Matrix factorization incorporating prior knowledge

In signal processing, various matrix factorization techniques have been developed that employ intrinsic properties of data to decompose them into underlying sources [17,18,20]. These methods are based on *delayed correlations *that can be defined for data having a temporal or spatial structure. For instance, the *time-delayed correlation matrix *of a centered, wide-sense stationary multivariate random process **x**(*t*) is defined as

(1) $${\left({\text{C}}_{\text{x}}(\tau )\right)}_{ij}:=E({\text{x}}_{i}(t+\tau ){\text{x}}_{j}{\left(t\right)}^{\text{T}}),$$

where E denotes expectation. Here, off-diagonal elements detect time-shifted correlations between different data dimensions. For *τ *= 0 this measure reduces to the common cross-correlation. Given *l *features, e.g. genes, aggregated in a data matrix **X**, e.g. mRNA expression data, the cross-correlation matrix can be easily estimated with the unbiased variance estimator:

(2) $${\text{C}}_{\text{X}}=\frac{1}{l-1}\text{X}{\text{X}}^{\text{T}}.$$

However, the experimentally generated quantitative data sets we face in bioinformatics rarely imply a natural order like which allows defining a generic kind of delayed correlation. We therefore generalize this concept by introducing prior knowledge that links features (e.g genes) along a pre-defined underlying network. This network may be large-scale, but can be also an explicitly given small-scale process. Moreover, integrated information may be of qualitative (e.g. interaction) as well as quantitative nature (e.g. interaction strength, reaction rates).

#### Graph-delayed correlation

We encode prior knowledge in a directed, weighted graph G:=(V,ℰ,w) defined on vertices V∈{1,…,l} corresponding to our features. The edges ℰ are weighted with weights *w*: ℰ → ℛ. These are collected in a *weight matrix ***W **∈ ℛ^*l*×*l*^, where *w_ij _*specifies the weight of edge *i *→ *j*. Note that our weights may be negative, and *G *may contain self-loops. For any vertex i∈V, we denote by *S*(*i*) := {*j*|(*i*, *j*) ∈ ℰ} the set of *successors of i*, by *P*(*i*) := {*j*|(*j*, *i*) ∈ ℰ} its *predecessors*.

The graph *G *introduces a partial ordering on the *l *features. We use the weight matrix **W **as propagator for an activity pattern X ∈ ℛ^*l *^of our features and define the *G-shift ***x**^*G *^of **x **as the vector with components

(3) $$x_{i}^{G}:=\sum_{j\in P(i)}{\text{W}}_{ji}x_{j}.$$

**Figure 2:**
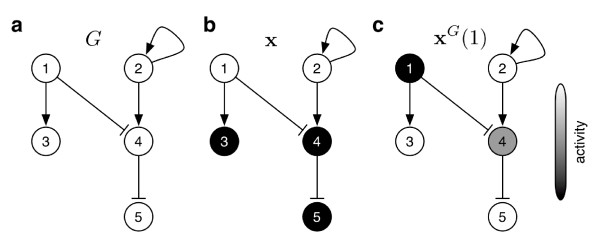
**Illustration of the *G*-shift**. Illustration of the *G*-shift in the unweighted graph *G *shown in (**a**). We start with an initial node activity **x **depicted in (**b**). We use the graph as propagator for the time evolution of this pattern: after one positive shift we achieve the activity pattern x^*G*^(1) in (**c**).

Recursively, we define any positive shift **x**^*G*^(*τ *) (see Figure 2). For negative shifts we replace predecessors *P*(*i*) by successors *S*(*i*), which formally corresponds to a transposition of the weight matrix **W**. Using the convention of trivial weights for non-existing edges of *G*, we can extend the above sum to all vertices. Gathering available m experiments (rows) into a data matrix **X **∈ ℛ^*m *× *l *^, we obtain the simple, convenient formulation of a G-shifted data set

(4) $${\text{X}}^{G}(\tau )=\{\begin{array}{cc} \text{X}{\text{W}}^{\tau } & \tau \geq 0\\ \text{X}{\left({\text{W}}^{\text{T}}\right)}^{\tau } & \tau <0 \end{array}.$$

After mean removal, we may assume that each row of **X **is centered. Then, in analogy to the unbiased estimator for cross-correlations in Equation 2, we define the *graph-delayed (cross)-correlation*

(5) $${\text{C}}_{\text{X}}^{G}(\tau ):=\frac{1}{l-1}{\text{X}}^{G}(\tau ){\text{X}}^{\text{T}}=\frac{1}{l-1}(\text{X}{\text{W}}^{\tau }{\text{X}}^{\text{T}}).$$

Note that our definition includes the standard time-delayed correlation by shifting along the line graph 1 → 2 → ... → *l - *1 → *l*.

The graph-delayed correlation is only symmetric if the used graph shows this feature which is, for instance in regulatory networks, rarely the case. For our following derivations, a symmetric generalized correlation measure however will turn out to be very convenient. In the remainder of this work, we will therefore use the *symmetrized graph-delayed correlation*

(6) $${\bar{\text{C}}}_{\text{X}}^{G}(\tau )=\frac{1}{2}({\text{C}}_{\text{X}}^{G}(\tau )+{\text{C}}_{\text{X}}^{G}{\left(\tau \right)}^{\text{T}}).$$

Enforcing the symmetry property is strategy has been often applied in the case of temporally or spatially delayed correlations. It has also been demonstrated that symmetrization stabilizes the estimation of the cross-correlations from data [8]. Moreover, it can be shown that asymptotically using either normal or symmetrized correlations end up giving the same eigenvectors [17].

#### Factorization model

The linear mixing model for the input data matrix **X **∈ ℛ^*m*×*l *^is given by

(7) X=AS+ε.

Here, the matrix of source contributions **A **∈ ℛ^*m*×*n *^(*m *≥ *n*) is assumed to have full column rank. The sources **S **∈ ℛ^*n*×*l*^are uncorrelated, zero-mean stationary processes with nonsingular covariance matrix. We allow for a noise term *ε *∈ ℛ^*m*×*l*^, which is modeled by a stationary, white zero-mean process with variance *σ*^2 ^. We assume white unperturbed data $\tilde{\text{X}}:=\text{A}\text{S}$ (possibly after whitening transformation). In other words, we interpret each row of **X **as linear mixture of the *n *sources (rows of **S**), weighted by mixing coefficients stored in **A**. Without additional restrictions, this general linear blind source-separation problem is underdetermined.

Here, we assume that the sources have vanishing graph-delayed cross-correlation with respect to some given graph *G *and all shifts *τ*. Formally, this means that ${\bar{\text{C}}}_{\text{S}}^{G}(\tau )$ is diagonal. We observe

(8) $${\bar{\text{C}}}_{\text{X}}^{G}(\tau )=\{\begin{array}{cc} \text{A}{\bar{\text{C}}}_{\text{S}}^{G}(\tau ){\text{A}}^{\text{T}}+\sigma^{2}\text{I}, & \tau =0\\ \text{A}{\bar{\text{C}}}_{\text{S}}^{G}(\tau ){\text{A}}^{\text{T}} & \tau \neq 0 \end{array}.$$

Clearly, a full identification of **A **and **S **is not possible, because Equation (7) defines them only up to scaling and permutation of columns: Multiplication of a source by a constant scalar can be compensated by dividing the corresponding row of the mixing matrix by the scalar. Similarly, the factorization implies no natural order of the sources. We can take advantage of the scaling indeterminacy by requiring our sources to have unit variance, i.e. ${\bar{\text{C}}}_{\text{S}}^{G}(0)=\text{I}$ With this, as we assumed white data $\tilde{\text{X}}$, we see that **AA**^T ^= I, i.e. **A **is orthogonal. Thus, the factorization in Equation (8) represents an eigenvalue decomposition of the symmetric matrix ${\bar{\text{C}}}_{\text{X}}^{G}(\tau )$. If additionally we assume that ${\bar{\text{C}}}_{\text{S}}^{G}(\tau )$ has pairwise different eigenvalues, the spectral theorem guarantees that **A **- and with it **S **- is uniquely determined by **X **except for permutation. The reason why we focused on the symmetrized instead of the simple graph-delayed correlation matrix was precisely that we wanted to have a symmetric matrix, because then the eigenvalue decomposition is well defined and also simple to compute.

However, we have to be careful, because we cannot expect ${\bar{\text{C}}}_{\text{X}}^{G}(\tau )$ to be of full rank. Obviously, we require more features than obtained sources (*l *» *m*), hence in general rank(**X**) = *m*. If *G *contains an adequate amount of information, rank(**W**) is of order *l *and since *l *» *m*, rank $\left({\bar{\text{C}}}_{\text{X}}^{G}(\tau )\right)$ is essentially determined by (the upper bound) *m*. Hence, when analyzing high-throughput biological data linked by underlying large-scale networks, we can extract as many sources as observations are available.

#### The GraDe algorithm

Equation (8) also gives an indication of how to solve the matrix factorization task in our setting. The first step consists of whitening the no-noise term $\tilde{\text{X}}=\text{A}\text{S}$ of the observed mixtures **X**. The whitening matrix can be easily estimated from **X **by diagonalization of the symmetric matrix ${\bar{\text{C}}}_{\tilde{\text{X}}}^{G}(0)={\bar{\text{C}}}_{\text{X}}^{G}(0)-\sigma^{2}\text{I}$, provided that the noise variance *σ*^2 ^is known or reasonably estimated. If more signals than sources are observed, dimension reduction can be performed in this step. Insignificant eigenvalues then allow estimation of the noise variance, compare [17]. Now, we may estimate the sources by diagonalization of the single, symmetric graph-delayed correlation matrix ${\bar{\text{C}}}_{\text{X}}^{G}(\tau )$. Altogether, we subsume this procedure as GraDe algorithm. In summary, the input of GraDe is *(i) *a expression matrix **X **∈ ℛ^*m*×*l *^containing m experiments and l genes and *(ii) *a *weight matrix ***W **∈ ℛ^*l*×*l *^containing the prior knowledge. We obtain a mixing matrix **A **∈ ℛ^*m*×*n *^(*m *≥ *n*) and a source matrix **S **∈ ℛ^*n*×*l *^. In the case of gene expression data analysis the sources can be interpreted as biological processes and the mixing coefficients as their time-dependent activities. A Matlab implementation is freely available at http://cmb.helmholtz-muenchen.de/grade.

Including prior knowledge into the source-separation task may introduce bias in the patterns that are pre-defined and, in turn, the analysis and results obtained. It is important to note that annotation of biological knowledge is biased and under permanent change. Therefore, when using gene regulatory networks as prior knowledge one has to keep in mind that this information is subject to annotation bias. Thus the density of connections in certain regions of the network might be higher due to the fact that these parts are better explored. In the case of classification problem, recent studies have shown that methods can be improved in terms of classification accuracy by including prior knowledge into the classification process [21]. These methods benefit from the fact that genes are not treated as independent. Hence, most of these methods are based on the hypothesis that genes in close proximity, which are connected to each other, should have similar expression profiles. The same assumption may be transferred to source-separation methods. Applying standard methods like ICA or PCA, implies the assumption that all data points, i.e. in our setting the expression levels of different genes are sampled i.i.d. from an underlying probability density. This assumption is obviously not fulfilled, since the genes' expression values are the read-outs of different states of a complex dynamical system: Genes obey dynamics along a transcription factor network. Instead of ignoring the genes' dependencies, we here proposed to explicitly model them using prior knowledge given within a gene-regulatory network. Therefore, one of the key advantages of GraDe is to overcome the assumption of the independencies. Applying GraDe to time-course expression data (see section *Validation of the time-dependent signals*), we will show that including prior knowledge into the source separation task leads to an improvement compared to fully-blind methods like PCA. Finally, we believe that with increasing quality and amount of biological knowledge, methods incorporating prior knowledge will increase in performance as well.

### Illustration of GraDe

**Figure 3:**
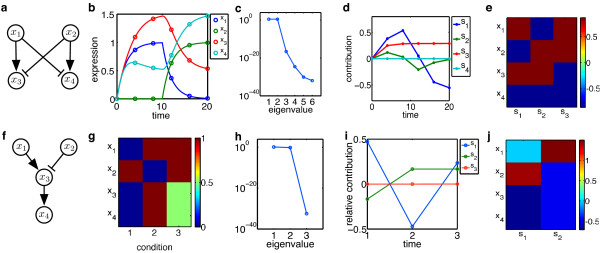
**Illustration of GraDe**. For the bifan motif in **a **we take 6 genes (dots) from the simulated time-courses in **b **(for parameters see Additional file 1) and apply GraDe: **c **shows the eigenvalues of the decomposition in GraDe. In **d **we plot the time-courses of the extracted sources *s*_1 ... _*s*_6_, hence the curves are the columns of the mixing matrix. From **c **we see that only the first three sources are relevant, which are visualized as heat-map **e**. For our second example **f **we assume to know expressions in different conditions as shown in **g**. The factorization by GraDe is visualized in sub figures **h **to **j**.

In order to illustrate GraDe, we analyze two toy examples. We first focus on a bifan structure shown in Figure 3a and assume to have six genes from the time-courses of expression levels depicted in Figure 3b. For data generation, the system is simulated by ordinary differential equations:

(9) $$\frac{\text{d}x_{i}(t)}{\text{d}t}=-\gamma_{i}x_{i}(t)+\sum_{j\in P(i)}f_{ji}(x_{j}(t)).$$

where we model interactions by sigmoidal Hill functions [22]. In this case, one input x_1 _is active until time-point 10, when it is turned off and instead production of x_2 _is switched on. Consequently, x_3 _peaks at time 10, but also x_4 _shows an early activation due to low expression of its inhibitor. Applying GraDe (with the known bifan topology, but without access to the underlying ODE system), we find that three sources are sufficient to explain the data (Figure 3b). From the extracted sources and their time-courses (shown in Figure 3e and 3d) we see that the strongest source s_1 _represents the externally controlled inputs and the network topology: the source couples *x*_1 _and *x*_3_, and in opposite direction *x*_2 _and *x*_4_. Therefore, GraDe is able to recover the two processes. Source *s*_2 _has the lowest contribution to the total expression values and is needed for fine-tuning the combined dynamics, as we obtain an early activation of *x*_4 _due to low expression of its inhibitor. Consequently, the source *s*_2 _is active at time-points 2 and 4, i.e. immediately after the switching operations. Source *s*_3 _again reflects the crossover inhibitions, accordingly its time-course is at. This source groups the input of the network, which could be linked e.g. to pathway stimulation. For our second example we use the funnel structure in Figure 3f, where we defined the expression values for three different input conditions (Figure 3g). Eigenvalues and the factorization obtained by GraDe are visualized in Figure 3h-j. Source *s*_1 _again reflects the network topology, by grouping the cascade genes, while s_2 _allows the reconstruction of the last condition. As we expect, GraDe are able to recover the two independent inputs. Applying GraDe to two different toy examples, we are able to show that GraDe is applicable both time-course as well as conditional experiments. In both cases, GraDe identifies the different responses and inputs of the system.

### Application: IL-6 mediated responses in primary hepatocytes

In liver, the cytokine interleukin *IL-6 *mediates two major responses. First, it induces hepatocytes to produce acute phase proteins upon infection-associated in inflammation. These proteins include complement factors to destroy or inhibit growth of microbes. In addition, *IL-6 *promotes liver regeneration and protects against liver injury [19]. *IL-6 *regulates several cellular processes such as proliferation, differentiation and the synthesis of acute phase proteins [23]. Upon binding to its cell surface receptor, *IL-6 *activates the receptor associated Janus tyrosine kinase (JAK) 1 - signal transducer and activator of transcription (STAT) 3 - signal transduction pathway. The latent transcription factor STAT3 is translocated to the nucleus after activation and subsequently alters gene expression.

To identify the biological responses to *IL-6 *in a time-resolved manner, we stimulated primary mouse hepatocytes with 1 nM *IL-6 *up to 4 hours and analyzed the changes in gene expression by microarray analysis. In a first approach, we extracted all genes that were significantly regulated compared to time point 0 h. In total, we obtained 121 genes and applied *k*-means clustering to detect groups within this set. Based on this approach, we could not identify any time-resolved responses upon *IL-6 *stimulation (see Additional file section *Clustering of significantly regulated genes*). Due to the small number of significantly regulated genes, we decided to employ a genome-wide approach using GraDe to resolve the cellular responses upon *IL-6 *in more detail.

#### GraDe discovers time-dependent biological processes upon IL-6 stimulation

**Figure 4:**
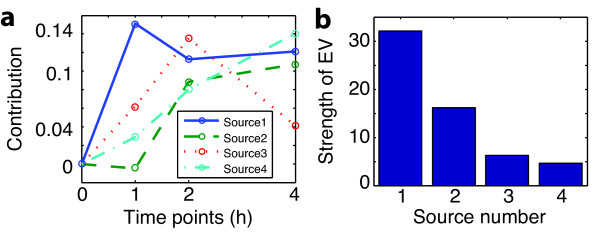
**Result of GraDe**. This figure illustrates the decomposition of the time-course microarray experiment on *IL-6 *stimulated hepatocytes with GraDe. As underlying network we used interactions from the TRANSPATH database (see Methods). (**a**) shows the time-courses of the four extracted sources, centered to time point 0 h. The *x*-axis shows the measured time-points and the *y*-axis the contribution of the mixing matrix. In (**b**), we plot the strength of the eigenvalues (EV) of the resulting sources. All four extracted sources have significant contributions.

Using GraDe, we linked all 5709 expressed genes along a gene regulatory network derived from the TRANSPATH database (see Methods). We obtained four graph-decorrelated gene expression sources (GES), which we labeled from 1 to 4 according to their decreasing eigenvalues (Figure 4b). We see that dimension reduction and with it noise level estimation were not possible in our case. The estimated mixing matrix is shown in Figure 4a. The matrix of source contributions contains positive and negative components. We partitioned a source into submodes that contain either the negative signals or the positive signals, respectively. We selected all genes in the positive submodes by choosing a threshold ≥2 as well as all genes in the negative submodes with a threshold ≤-2, respectively. These sets were subsequently used for GO enrichment analysis using a *p*-value < 0.05 after correction by False Discovery Rate.

Differentially expressed genes within GES 1 display an immediate strong increase in expression following *IL-6 *stimulation. After peaking at one hour, expression decreases to elevated levels compared to untreated samples. Significantly enriched GO-Terms within this GES correspond to responses triggered by external stimuli and in ammation (see Table 1, for a complete list of biological processes see Additional file 1). In liver, upon infection- or injury-associated in inflammation *IL-6 *mediates production of acute phase proteins (APP) by hepatocytes as represented by the GO-Term "(acute) in ammatory response " (e.g *Saa4*, *Fgg*, *Pai1 *). *Angptl4 *is a positive acute phase protein [24] showing a strong increase in expression during the first hour after stimulation followed by a decrease after two hours (see Figure S4). GraDe reconstruct the expression pattern by the mixing of the four different source time-patterns GES 1 to 4. We identify *Angptl4 *in GES 1 and 3 having a source contribution ≥2. The combination of both GESs showing perfectly the strong increase after *IL-6 *(GES 1) and the induced decreased after 2 hours (GES 3). The GO-Term "(external) stimulus" includes genes of the JAK-STAT signaling pathway like *STAT3 *as well as several genes encoding for signaling components such as *Hamp*, *Cepbd *and *Osmr*. These entities represent regulatory processes like negative feedbacks as well as secondary signaling events. Genes with negative contribution in GES 1 were associated with metabolic processes like "L-serine biosynthesis" or "fructose metabolic processes". This is in line with the function of *IL-6 *as a priming factor, mediating the conversion of quiescent hepatocytes from G0 to G1 phase of the cell cycle during liver regeneration [19]. It can be argued that down-regulation of genes associated with metabolic processes is due to the transformation of differentiated metabolically active hepatocytes into proliferative cells. The down-regulated metabolic functions at least partially take place in mitochondria. Accordingly, parts of the glycolysis pathway were down-regulated in primary hepatocytes.

**Table 1:** Main biological processes in response to IL-6

Source	Mode	Biological process
1	positive	(external) stimulus, inflammatory response
	negative	(fructose) metabolic process
2	positive	early cell cycle and division
	negative	metabolic process, apoptosis
3	positive	late cell cycle and division
	negative	-
4	positive	translation, coagulation
	negative	(protein) metabolic process

Summary of the main biological processes in hepatocytes regulated as response to *IL-6*. Mode indicates genes with significant positive (≥2) or negative (≤-2) contribution to the source. The main biological processes found for the corresponding group of genes are given in the last column.

GES 2 shows a slight decrease after stimulation followed by a late-phase increase in expression. We identify several biological processes associated with "cell cycle and division" within this GES. A representative gene of GES 2 is the cell cycle inhibitor *Cdkn1b*. Its reduction of expression corresponds to the induction of cell cycle progression and in particular to the transfer from G0 to G1. These characteristics are further supported by the negative contribution of *Cdkn1b *in GES 3. Analyzing genes with a positive contribution in GES 2 only, we found, in addition to involvement in early cell cycle events, genes showing an association with (programmed) cell death and apoptosis. It was already indicated that *IL-6 *promotes liver regeneration and protects against liver injury by inducing anti-apoptotic and survival genes [19,25]. GO-Terms corresponding to genes found in GES 2 having a negative contribution are more heterogeneous. Within the top GO-Terms we identified several biological functions associated with the *IL-6 *stimulus. Based on the induction of the acute in ammatory response, coagulation factors were activated. Moreover, several genes associated with gene translation were found. In addition, genes associated with metabolic processes are represented by this GES.

The time course behavior of GES 3 shows a delayed activation subsequent to stimulation with *IL-6*. We identified several GO-Terms associated with "cell cycle" and "cell division" similar to GES 2. However, GES 3 includes mainly genes related to late events in the cell cycle, i.e. during G2 and M phase (e.g. *Gmnn*, *Mcm2*, *Plk2 *). *Wee1 *as a main regulator of *Cdc2 *displays a negative contribution to GES 3, hence indicating *Wee1 *down-regulation and subsequent progression through the G2-M check point. In addition, we identify *Ccnb2 *a late cell cycle genes, which repression leads to cell cycle arrest in the G2 phase. The time-course expression pattern, shows a strong increase after *IL-6 *stimulation followed by a decrease after two hours (see Figure S5). We identify *Ccnb2 *in GES 1 and GES 3 perfectly reconstruct the strong increase after the stimulation and the inactivation after two hours. The *IL-6 *-induced priming phase is characterized by the activation of the latent transcription factor *STAT3*. This immediate response induces the expression of early responsive genes like the transcription factor *AP-1 *[26] subsequently inducing a secondary gene response leading to transcription of cyclins *A-E*, *p53*, and the cyclin dependent kinase *P34-cdc2 *[27].

Applying KEGG pathway enrichment, we found the cell cycle, with DNA replication in particular, and p53 pathway enriched within this GES. Interestingly, *IL-6 *stimulation alone is not sufficient to efficiently induce proliferation of primary mouse hepatocytes *in vitro*. Hence, despite the persistent re-organization of the induced gene expression profile and the induction of early cell cycle players such as cyclin *A*, additional stimuli may be necessary to initiate a strong proliferative response of primary mouse hepatocytes. GES 4 shows the lowest eigenvalue. It has a strong increase in expression following the *IL-6 *stimulus. GO-Term enrichment reveals several biological processes found in GES 1 - 3 like coagulation, translation, acute phase, and response of the stimulus. Genes having a negative contribution in GES 4, indicating a decrease in expression after the stimulus, are again associated with metabolic processes. Both, GES 3 and 4 imply that hepatocytes stimulated with *IL-6 *show affection for division causing a down-regulation of genes associated with the metabolic processes.

### Validation of the time-dependent signals

In order to evaluate our findings, we compared the outcome of GraDe with standard methods. As there is no established matrix factorization technique that incorporates prior knowledge, we employed PCA [28], *k*-means clustering [29] and FunCluster [30], a clustering method that incorporates Gene Ontology information into the clustering task.

To test the biological findings obtained by GraDe, we applied a similar approach as proposed by Teschendor *et al*. [12]. We first asked how well biological pathways can be mapped to the inferred submodes or clusters. For GraDe and PCA, we selected in each submode the genes having an absolute source contribution above 2 standard-deviations. The average number of selected genes in each submode ranges from 75 to 280. For *k*-means clustering, we infer 8 clusters on a subset of the top 15% most variable genes to ensure that the average number of selected genes is comparable to GraDe and PCA.

**Figure 5:**
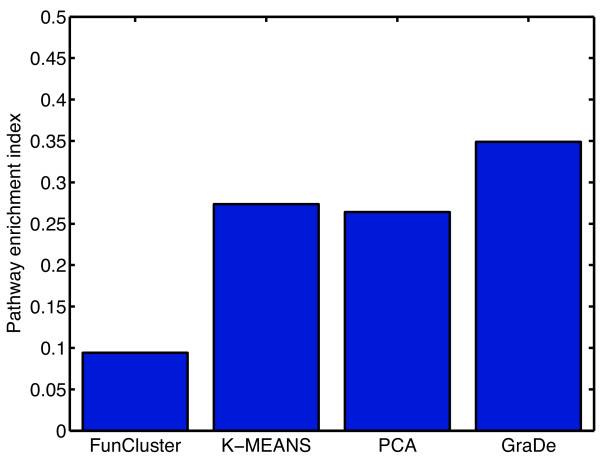
**Pathway enrichment**. Result of the pathway enrichment analysis. For each method applied to our data set, we plotted the pathway enrichment index (PEI). This index gives the fraction of KEGG pathways found enriched in at least one submode or cluster (see Methods). GraDe obtained a much higher PEI than PCA, *k*-means clustering or FunCluster. This indicates that sources obtained by GraDe map much closer to biological pathways.

To evaluate the mapping of pathways to submodes or clusters we applied the pathway enrichment index (PEI). For each submode or cluster we evaluated significantly enriched pathways by using a hypergeometric test (see Methods). The PEI is then defined as the fraction of significant pathways mapped to at least one submode or cluster. The PEI for each method is shown in Figure 5. We find that the PEI is higher for GraDe compared to PCA, *k*-means clustering or FunCluster indicating that GraDe maps submodes closer to biological pathways.

**Figure 6:**
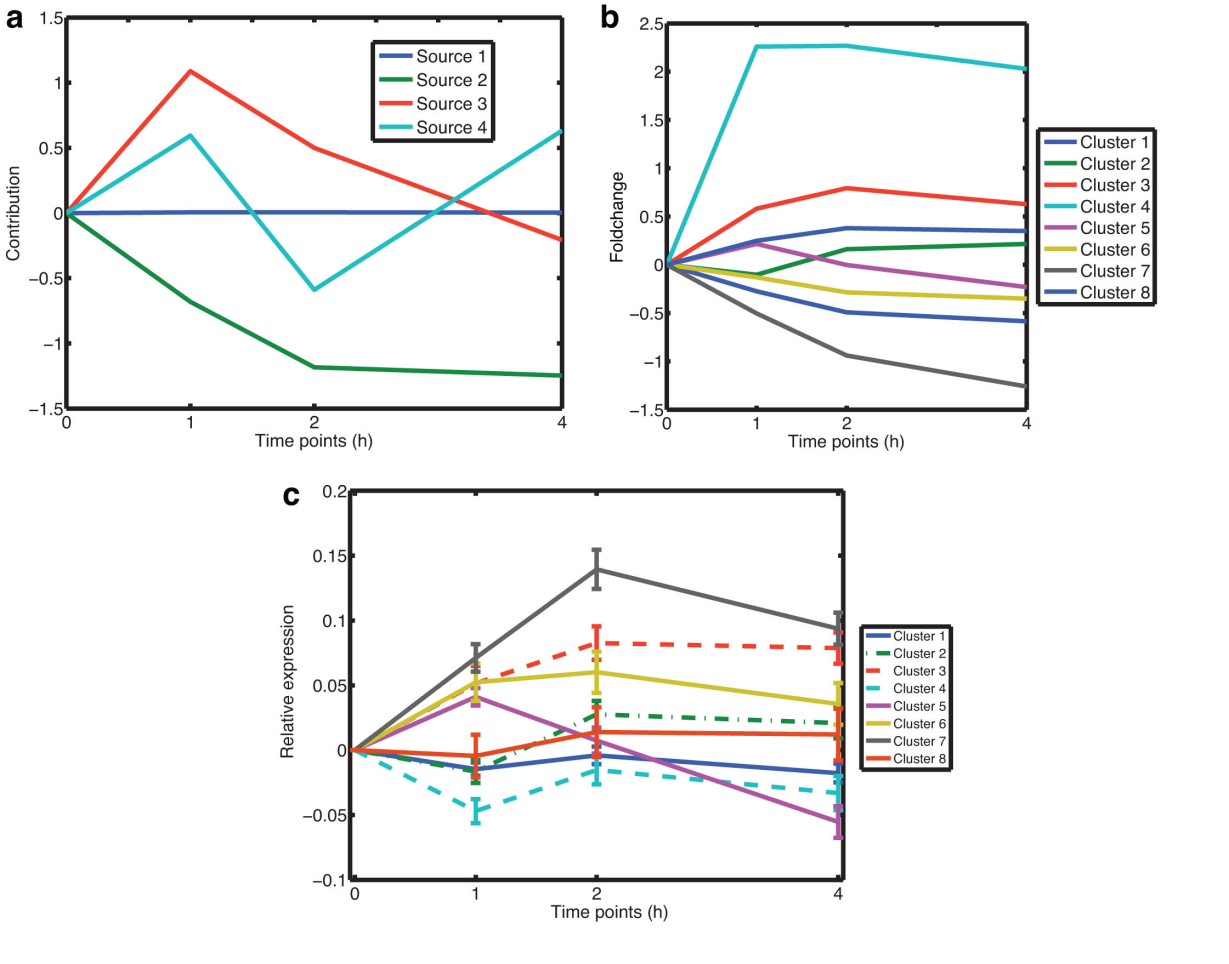
**Result of PCA, *k*-means clustering and FunCluster**. (**a**) illustrates the result of PCA for the time-course data of *IL-6 *stimulated hepatocytes. The *x*-axis corresponds to the measured time-points and the *y*-axis gives the centered (to time point 0 h) contributions of the mixing matrix. The result of the *k*-means clustering is shown in (**b**). The *x*-axis shows the measured time-points and the *y*-axis shows the fold-change values of the centroids at that time-points. (**c**) shows the result of FunCluster. The plot shows the mean expression of the different cluster and the bars indicates the standard deviation at a particular time-point. The *x*-axis shows the measured time-points and the *y*-axis shows the relative expression at that time-points.

In addition, we validated the time-dependent responses upon *IL-6 *stimulation in more detail by searching for enriched GO-Terms. Applying PCA, we found that the first principle component (PC) contains 99% of data variance (see Figure S1). GO-Term enrichment analysis revealed that PC 1 contains genes linked to (blood) coagulation and hemostasis (see Additional file 1). A second major response after *IL-6 *is the activation of cell cycle or cell division. We found an enrichment of these biological processes in PC 2 and PC 4. PC 2 shows a decreased time-course behavior after the stimulation. Genes linked to cell cycle and corresponding pathways have a negative contribution in PC 2 indicating an increased time-course expression pattern after IL-6 stimulation. This finding is analogous to GraDe, where we find cell-cycle pathways in GES 1 and 3 showing also an increasing expression pattern after the stimulation. With GraDe we identified several genes that are associated with metabolic processes showing a down-regulation after stimulus. PCA covers these biological processes by two components PC 2 and PC 3, where PC 3 shows a strong increase and PC 2 a decrease of expression after the stimulus (see Figure 6a). The direct response of *IL-6 *was found in PC 4, but we identified only acute in ammatory response. Moreover, PCA grouped cell cycle (negative mode) and the direct response (positive mode) into PC 4 and was not able to separate the cell cycle processes into the early (e.g. *Cdkn1b*) and late (e.g *Mcm2 *) responses after *IL-6 *stimulation.

Focusing on the results of the *k*-means clustering, we obtained an enrichment of cell cycle processes in cluster 3 (see Figure 6b). This cluster shows only a marginal increase in expression after the stimulus and therefore does not reflect the strong activation of cell cycle found by GraDe and PCA. Genes associated with metabolic processes are grouped in cluster 5, which has a constant expression level after *IL-6 *stimulus. Hence, *k*-means clustering failed to infer a cluster associated to the downregulation of metabolic processes upon *IL-6*. Cluster 4 shows a characteristic time-course pattern after *IL-6 *stimulation, but we were not able to reveal any significant biological processes associated to *IL-6*. Altogether, *k*-means clustering neither identifies the direct response upon *IL-6 *nor the separation between early and late cell cycle genes. Comparing the result of FunCluster, we also identify a set of co-regulated genes associated with cell cycle (Cluster 3; see Figure 6c). Genes grouped in this cluster show an increase in expression after the stimulus. However, FunCluster was also not able to separate the early and late cell cycle processes, observed by GraDe. Genes associated with metabolic processes are grouped in cluster 5, showing a decreasing expression pattern after one hour of stimulation. Therefore, FunCluster also identifies the downregulation of metabolic processes indicating that IL-6 reduces expenditures for the energy metabolism. However, FunCluster was not able to identify the primary response of IL-6 mediating the production of acute phase proteins (APP) by hepatocytes. Moreover, FunCluster also did not find any significant processes related to the JAK-STAT related genes, such as *Stat3*, *Hamp*, *Cepbd *and *Osmr*, showing an increased expression pattern.

These results show that the decomposition obtained by GraDe provided much more detailed biological insights than PCA, *k*-means clustering or FunCluster. PCA was able to identify three main biological processes upon *IL-6 *stimulus. However, it failed to give a correct time-resolved pattern of these biological processes, whereas sources from GraDe reproduce the characteristic time-course behavior of the *IL-6 *response. Moreover, GraDe reveals a much more structured and time-resolved result, which allows assigning each source to a different main process.

### Robustness analysis

**Figure 7:**
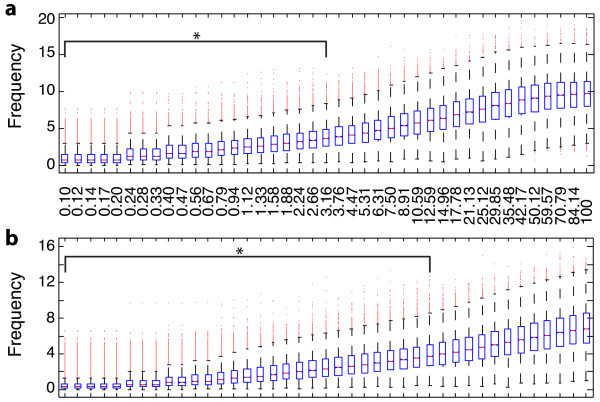
**Robustness analysis**. Robustness analysis: We evaluated the robustness of GraDe against errors in the underlying graph. To this end, we compared the mixing matrix that we extracted with the TRANSPATH network with those obtained based on perturbed versions. For this comparison we use the Amari index (see Methods). The boxplots show Amari-indices obtained with (**a**) a network rewiring approach and (**b**) when adding random information to the network. The x-axis shows the amount of information randomized (in %), the y-axis gives the obtained Amari-index. * indicates significant 95% quantiles compared to a random sampling (*p*-value ≤ 0.05). We see that GraDe is robust against a reasonable amount of wrong information.

Detailed knowledge about gene regulation is often not available and far from complete. Therefore, the quality of a large-scale gene regulatory network is not perfect. In order to test the effect of network errors on the output of GraDe, we performed two robustness analyses. Starting with our TRANSPATH network, we generated randomized versions by either shuffling the network content or adding random information (see Methods). By shuffling edge information of the gene regulatory network between 0.1 and 100% of all original edges, we simulated a loss of information. To quantify robustness, we employed the Amari-index, which measures the deviation between two mixing matrices. We obtained significantly low Amari-indices for up to 3% reshuffled edges within the gene regulatory networks (mean Amari-index = 3.83, *p *= 0:034), whereas a complete randomization of the network results in an Amari-index of 9.63 (see Figure 7a). This shows that the quality of the regulatory network has of course a strong influence on the output of the GraDe algorithm. It is obvious that GraDe depends on the regulatory network, and replacing gene interaction through random information will lead to loss of the signals.

We ran a second robustness analysis by adding random information to the existing gene regulatory network. This is important because we expect large-scale networks extracted from literature to contain many false-positives. Significantly low Amari-indices were obtained by adding up to 13% random information (mean Amari-index = 3.94, *p *= 0:046) to the network (see Figure 7b). This result shows that GraDe is able to detect the signals even after adding a large amount of probably wrong information to the network. The tolerance of the algorithm to the second randomization strategy is much higher, as here no correct information is destroyed. Overall, with both randomization procedures we were able to prove that GraDe is robust against a reasonable amount of both, false positives and missing information.

In addition, we analyzed the noise effect of gene expression data by randomly choosing between one and three replicates for each time point. We found significantly low Amari-indices (mean Amari-index = 4.16 *p *= 0:026) by comparing the 95% quantile of the resulting Amari-index with a random sampling. Thus, GraDe is also robust against biological noise.

## Conclusion

*IL-6 *promotes liver regeneration and protects against liver injury. In order to understand these effects in a time-resolved manner, we performed a time-course microarray experiment of *IL-6 *stimulated primary mouse hepatocytes. Standard techniques applied to this data set only partly revealed temporal gene expression patterns following the stimulation. To resolve the interaction of *IL-6 *and the corresponding cellular responses in more detail, we developed GraDe. It extracts overlapping clusters from large-scale biological data by combining a matrix factorization approach with the integration of prior knowledge. Applying GraDe to our experiment, we identified the activation of acute phase proteins, which are known to be one of the primary response upon infection based in ammation. Moreover, we observed that *IL-6 *activates cell cycle progression, as well as the down-regulation of genes associated with metabolic processes and programmed cell death. Therefore, *IL-6 *mediated priming renders hepatocytes more responsive towards cell proliferation and reduces expenditures for the energy metabolism.

## Methods

### IL-6 stimulated mouse hepatocytes

RNA probes from primary mouse hepatocytes were assessed with the Bioanalyzer 2100 (Agilent) to ensure that 28S/18S rRNA ratios were in the range of 1.5 to 2.0 and concentrations were comparable between probes. For each time point, 4 g of total RNA were used for the hybridization procedure using the One-Cycle Target Labeling Kit (Affymetrix). Fluorescence intensities were acquired with the GeneChip Scanner 3000 and the GCOS software (Affymetrix). GeneChip Mouse Genome 430 2.0 Arrays (Affymetrix) were used in the analysis comprising stimulations with 1 nM *IL-6 *for 1 h, 2 h, 4 h and an unstimulated control (0 h) each performed in triplicates. As a probe level model (PLM) for microarray data an additive-multiplicative error model was used. Data processing was performed using the Limma toolbox [31] provided by Bioconductor [32]. The RMA approach was used for normalization and background correction. Probe sets were filtered out by the genefilter package. A gene was considered as expressed if the signal was above 100 (unlogged data) for at least one time point. Finally, we obtained a data set of 5709 genes. Significantly regulated genes compared to time point 0 h were determined by using the LIMMA (Linear Models for Microarray Data) method [33]. The Limma toolbox uses the moderated *t*-statistics to identify significant regulated genes. Moreover the moderated *t*-statistics is advisable for a small number of arrays [33,34]. A gene was determined as significant regulated if the *p*-value was <0.05 after multiple testing correction by the Benjamini-Hochberg procedure [35]. Raw data are available at GEO with accession number GSE21031.

### Gene Regulatory network

In order to link genes along an underlying network we used the TRANSPATH database [36] that provides detailed knowledge of intracellular signaling information based on changes in transcription factor activity.

We searched for direct gene or protein interactions within the TRANSPATH database using the terms: transactivation, increase of abundance, expression, activation, DNA binding, increase of DNA binding, transrepression, decrease of abundance, decrease of DNA binding, and inhibition.

### Principle component analysis

For principle component analysis (PCA) we performed an eigenvalue decomposition of the covariance matrix of the data set *X*. Thereby we obtained a decomposition into an orthonormal source matrix S and an orthogonal mixing matrix *A*. We applied PCA to the same set of expressed genes as GraDe and also inferred four sources. We defined for each component two submodes by grouping genes with a threshold ≥+2 standard-deviations and a second set of genes having a source weight of ≤-2 standard-deviations.

### *k*-means clustering

In order to ensure a fair comparison of *k*-means clustering with GraDe and PCA, we first applied a gene selection step to provide that all methods selected an approximately equal number of genes, as proposed in [12]. We ranked all expressed genes according to their expression variance across the time-course and then selected the top 15% variable genes. Having the selected genes, clustering was then performed using *k*-means clustering [29], where *k *was set to 8 in order to match the same number of submodes inferred by GraDe and PCA.

### FunCluster

In addition to *k*-Means clustering, we also include a clustering method which incorporates Gene Ontology information into the clustering task. We use the FunCluster method, which performs functional analysis of gene expression data [30,37]. FunCluster detects co-regulated biological processes through a specially designed clustering procedure involving biological annotations (GO and KEGG) and gene expression data. We apply the FunCluster implementation provided within the R environment [38] and using standard parameters.

### Enrichment analysis

For gene sets grouped in sources obtained by GraDe and PCA or *k*-means clusters we performed an enrichment analysis to determine significantly enriched biological processes and pathways. For biological processes we performed a Gene Ontology (GO) [39] term enrichment analysis, which was carried out with the R package GOstats [32]. For pathway enrichment analysis we used non-metabolic pathways that are manually curated by the Kyoto Encyclopedia of Genes and Genomes (KEGG) [40]. Pathway enrichment was also evaluated with the GOstats package. To correct for multiple testing, we used the Benjamini-Hochberg procedure [35] and called an association significant if the *p*-value was less than 0.05. To evaluate the mapping of pathways to submodes or clusters we applied the pathway enrichment index (PEI), as proposed by [12]. For each submode or cluster we evaluated the significance of enrichment of a set of genes in a particular pathway by using a hypergeometric test. A pathway association was considered as significant if the *p*-value was below 0.05 after multiple testing correction using the Benjamini-Hochberg procedure. The PEI was then defined as the fraction of significant pathway mapped to at least one submode or cluster.

### Robustness analysis

Robustness analysis was performed by two network randomizations. The gene regulatory network is interpreted as a weighted bipartite graph, i.e. a graph with two sets of nodes (regulators and regulated genes). Weighted edges indicate interactions either activating or inhibiting. First, we randomized existing edge information within the network between 0.1 and 100%. In each step we shuffled 10:000 times the corresponding amount of edges using a degree-preserving rewiring [41,42]. Applying GraDe with the resulting networks we obtained new factorizations. To compare the original and new results in a quantitative way, we used the Amari-index [43]. For each step we took the 95% quantile of the random sampling and calculated a *p*-value by comparing this quantile to Amari-indices obtained comparing normally distributed random separating matrices and the original mixing matrix. In a second randomization approach, we added 10:000 times new information (edges) between 0.1 and 100% of the original network content and calculated the 95% quantile of the resulting Amari-indices. Again, the *p*-value was calculated by comparing each quantile with a random sampling.

For analysis of robustness against noise we randomly chose between one and three replicates for each time point and ran GraDe. For each run, we calculated the Amari-index. Again, we compared the 95% quantile of the resulting distribution with a random sampling to obtain the corresponding *p*-value.

## Authors' contributions

FJT and UK conceptualized the study. AK, FB and FJT designed and implemented the algorithm, AK analyzed and interpreted the expression data. SB, MS and NG performed the experiment. AK, FB, SB, UK and FJT wrote the manuscript. All authors read and approved the final version of the manuscript.

## Supplementary Material

Additional file 1 **Supplementary information**. Additional file contains supplementary information. Click here for file
