# Supplementary material for: Knowledge-based matrix factorization temporally resolves the cellular responses to IL-6 stimulation
Source: BMC Bioinformatics. 2010 Nov 30;11:585. doi: 10.1186/1471-2105-11-585 (PMC3009690; doi:10.1186/1471-2105-11-585)
Supplement: Additional file 1 — Supplementary information. Additional file contains supplementary information. [file 1471-2105-11-585-S1.PDF]

Supplementary Information

Knowledge-based matrix factorization temporally resolves the cellular responses to IL-6 stimulation

Andreas Kowarsch[1], Florian Blöchl[1], Sebastian Bohl[2], Maria Saile[3], Norbert Gretz[3], Ursula Klingmüller[2], Fabian J. Theis[1,4]

[1] Institute for Bioinformatics and Systems Biology - MIPS, Helmholtz Zentrum München, Neuherberg, Germany

[2]Division of Systems Biology of Signal Transduction, DKFZ-ZMBH Alliance, German Cancer Research Center, Heidelberg, Germany

[3]Medical Research Center, Medical Faculty Mannheim, University Heidelberg, Mannheim, Germany

[4] Department of Mathematical Science, Technische Universität München, Garching, Germany

Table of contents

GraDe results **2**
Go-Term enrichment 2
Pathway enrichment 10

PCA results **14**
GO-Term enrichment 14
Pathway enrichment 21

Result of the k-means clustering **24**
GO-Term enrichment 24
Pathway enrichment 29

FunCluster results **32**
GO-Term enrichment 32
Pathway enrichment 35

Clustering of significantly regulated genes **36**

Yeast cell cycle data **40**

Illustration of GraDe **43**
Bifan topology 43
Funnel topology 43

Examples of overlapping clusters **44**

GraDe results

To analyze alterations in the gene response in *IL-6* stimulated primary mouse hepatocytes by GraDe, a time-course microarray experiment was performed. Based on the time-course gene expression data we observed 5709 genes expressed during the experiment. The gene list of the time-course expression data and the corresponding gene regulatory network can be downloaded on our webpage: http://cmb.helmholtz-muenchen.de/grade/. A complete list of all genes and the corresponding source contribution is also available on our webpage.

The following subsections list the significantly enriched GO–terms and pathways found in the source matrix obtained by GraDe.

GO-Term enrichment

Source 1

GOBPID	Term	Count	Size	p-value
GO:0006950	response to stress	27	979	0.0001
GO:0050896	response to stimulus	38	1775	0.0001
GO:0009611	response to wounding	14	313	0.0004
GO:0048008	platelet-derived growth factor receptor signaling pathway	4	18	0.0055
GO:0006952	defense response	13	360	0.0068
GO:0065007	biological regulation	68	5066	0.0124
GO:0009605	response to external stimulus	15	516	0.0131
GO:0002376	immune system process	18	707	0.0131
GO:0060325	face morphogenesis	3	11	0.0151
GO:0006954	inflammatory response	9	210	0.0169
GO:0060323	head morphogenesis	3	13	0.0211
GO:0042060	wound healing	6	94	0.0224
GO:0060324	face development	3	15	0.0280
GO:0002526	acute inflammatory response	5	71	0.0402
GO:0060322	head development	3	18	0.0402
GO:0051252	regulation of RNA metabolic process	21	1041	0.0402
GO:0010171	body morphogenesis	3	19	0.0402
GO:0043029	T cell homeostasis	3	19	0.0402
GO:0048513	organ development	27	1523	0.0402
GO:0040007	growth	10	323	0.0491

Table 1: GO–Term enrichment for all genes showing a positive contribution (≥ 2) in source 1. GOBPID shows the corresponding GO–Term ID. Term indicates the biological process. Count shows the number of genes found for this GO–Term and Size is the number of all genes for the corresponding GO–Term. The p-value is based on the conditional hypergeometric test, corrected by FDR.

GOBPID	Term	Count	Size	p-value
GO:0006002	fructose 6-phosphate metabolic process	2	2	0.0161
GO:0006564	L-serine biosynthetic process	2	3	0.0241
GO:0006000	fructose metabolic process	2	5	0.0442
GO:0050873	brown fat cell differentiation	3	27	0.0442
GO:0006563	L-serine metabolic process	2	6	0.0442
GO:0009070	serine family amino acid biosynthetic process	2	7	0.0442
GO:0048514	blood vessel morphogenesis	6	202	0.0442
GO:0045765	regulation of angiogenesis	3	32	0.0442

Table 2: GO–Term enrichment for all genes showing a negative contribution ($\leq$ -2) in source 1. GOBPID shows the corresponding GO–Term ID. Term indicates the biological process. Count shows the number of genes found for this GO–Term and Size is the number of all genes for the corresponding GO–Term. The p-value is based on the conditional hypergeometric test, corrected by FDR.

Source 2

GOBPID	Term	Count	Size	p-value
GO:0051301	cell division	10	261	0.0017
GO:0000278	mitotic cell cycle	9	249	0.0047
GO:0022402	cell cycle process	10	368	0.0095
GO:0007049	cell cycle	13	625	0.0095
GO:0022403	cell cycle phase	9	317	0.0095
GO:0000087	M phase of mitotic cell cycle	7	188	0.0095
GO:0000280	nuclear division	7	188	0.0095
GO:0007067	mitosis	7	188	0.0095
GO:0048285	organelle fission	7	192	0.0096
GO:0000279	M phase	8	279	0.0147

Table 3: GO–Term enrichment for all genes showing a positive contribution ($\geq$ 2) in source 2. GOBPID shows the corresponding GO–Term ID. Term indicates the biological process. Count shows the number of genes found for this GO–Term and Size is the number of all genes for the corresponding GO–Term. The p-value is based on the conditional hypergeometric test, corrected by FDR.

GOBPID	Term	Count	Size	p-value
GO:0006412	translation	35	301	5.8960e-17
GO:0055114	oxidation reduction	34	572	4.1070e-08
GO:0006082	organic acid metabolic process	29	436	4.1070e-08
GO:0019752	carboxylic acid metabolic process	29	436	4.1070e-08
GO:0043436	oxoacid metabolic process	29	436	4.1070e-08
GO:0042180	cellular ketone metabolic process	29	447	6.1731e-08
GO:0008202	steroid metabolic process	16	155	9.7800e-07

Table 4 continued on next page

GOBPID	Term	Count	Size	p-value
GO:0006869	lipid transport	13	96	9.7800e-07
GO:0044255	cellular lipid metabolic process	30	547	1.0539e-06
GO:0006629	lipid metabolic process	31	637	8.2922e-06
GO:0008152	metabolic process	144	6574	3.0478e-05
GO:0032787	monocarboxylic acid metabolic process	17	253	0.0001
GO:0006519	cellular amino acid and derivative metabolic process	17	263	0.0001
GO:0060192	negative regulation of lipase activity	3	3	0.0004
GO:0006775	fat-soluble vitamin metabolic process	6	28	0.0004
GO:0006066	alcohol metabolic process	18	319	0.0004
GO:0030300	regulation of intestinal cholesterol absorption	3	4	0.0011
GO:0032371	regulation of sterol transport	3	4	0.0011
GO:0032374	regulation of cholesterol transport	3	4	0.0011
GO:0009611	response to wounding	17	313	0.0011
GO:0009308	amine metabolic process	16	283	0.0012
GO:0008203	cholesterol metabolic process	8	72	0.0014
GO:0034097	response to cytokine stimulus	5	24	0.0021
GO:0032368	regulation of lipid transport	3	5	0.0021
GO:0044058	regulation of digestive system process	3	5	0.0021
GO:0051346	negative regulation of hydrolase activity	4	13	0.0021
GO:0016125	sterol metabolic process	8	79	0.0021
GO:0015918	sterol transport	5	25	0.0021
GO:0030301	cholesterol transport	5	25	0.0021
GO:0006720	isoprenoid metabolic process	6	41	0.0023
GO:0006575	cellular amino acid derivative metabolic process	10	129	0.0023
GO:0007596	blood coagulation	7	66	0.0042
GO:0019439	aromatic compound catabolic process	4	16	0.0042
GO:0050818	regulation of coagulation	4	16	0.0042
GO:0007599	hemostasis	7	67	0.0042
GO:0009063	cellular amino acid catabolic process	6	47	0.0042
GO:0044267	cellular protein metabolic process	53	1946	0.0042
GO:0009605	response to external stimulus	21	516	0.0042
GO:0016054	organic acid catabolic process	7	69	0.0042
GO:0046395	carboxylic acid catabolic process	7	69	0.0042
GO:0050817	coagulation	7	69	0.0042
GO:0031099	regeneration	4	17	0.0042
GO:0030299	intestinal cholesterol absorption	3	7	0.0042
GO:0042572	retinol metabolic process	3	7	0.0042
GO:0044241	lipid digestion	3	7	0.0042
GO:0042060	wound healing	8	94	0.0044
GO:0019538	protein metabolic process	61	2371	0.0044
GO:0002526	acute inflammatory response	7	71	0.0044
GO:0006766	vitamin metabolic process	7	71	0.0044
GO:0044237	cellular metabolic process	119	5626	0.0047
GO:0044238	primary metabolic process	122	5819	0.0051
GO:0006414	translational elongation	4	19	0.0057
GO:0009093	cysteine catabolic process	2	2	0.0062
GO:0019448	L-cysteine catabolic process	2	2	0.0062

Table 4 continued on next page

GOBPID	Term	Count	Size	p-value
GO:0019452	L-cysteine catabolic process to taurine	2	2	0.0062
GO:0046439	L-cysteine metabolic process	2	2	0.0062
GO:0042221	response to chemical stimulus	21	543	0.0062
GO:0009310	amine catabolic process	6	55	0.0063
GO:0050896	response to stimulus	48	1775	0.0075
GO:0006776	vitamin A metabolic process	4	21	0.0075
GO:0006950	response to stress	31	979	0.0077
GO:0043434	response to peptide hormone stimulus	6	58	0.0079
GO:0009725	response to hormone stimulus	7	82	0.0084
GO:0060191	regulation of lipase activity	4	22	0.0085
GO:0006520	cellular amino acid metabolic process	10	167	0.0092
GO:0044106	cellular amine metabolic process	10	167	0.0092
GO:0030193	regulation of blood coagulation	3	10	0.0092
GO:0060706	cell differentiation involved in embryonic placenta development	3	10	0.0092
GO:0050878	regulation of body fluid levels	7	86	0.0103
GO:0010033	response to organic substance	13	270	0.0105
GO:0001523	retinoid metabolic process	4	24	0.0105
GO:0016101	diterpenoid metabolic process	4	24	0.0105
GO:0034754	cellular hormone metabolic process	6	63	0.0105
GO:0034641	cellular nitrogen compound metabolic process	11	204	0.0105
GO:0044270	nitrogen compound catabolic process	6	64	0.0111
GO:0009074	aromatic amino acid family catabolic process	3	11	0.0111
GO:0006631	fatty acid metabolic process	10	175	0.0111
GO:0009058	biosynthetic process	75	3244	0.0111
GO:0006721	terpenoid metabolic process	4	25	0.0111
GO:0042632	cholesterol homeostasis	4	25	0.0111
GO:0055092	sterol homeostasis	4	25	0.0111
GO:0000098	sulfur amino acid catabolic process	2	3	0.0124
GO:0044273	sulfur compound catabolic process	2	3	0.0124
GO:0048583	regulation of response to stimulus	12	247	0.0133
GO:0044249	cellular biosynthetic process	73	3167	0.0142
GO:0006952	defense response	15	360	0.0150
GO:0006953	acute-phase response	4	28	0.0161
GO:0050892	intestinal absorption	3	13	0.0164
GO:0006725	cellular aromatic compound metabolic process	7	98	0.0172
GO:0006790	sulfur metabolic process	6	72	0.0172
GO:0043086	negative regulation of catalytic activity	6	72	0.0172
GO:0009719	response to endogenous stimulus	7	99	0.0179
GO:0006955	immune response	16	409	0.0183
GO:0045834	positive regulation of lipid metabolic process	3	14	0.0193
GO:0019530	taurine metabolic process	2	4	0.0212
GO:0033209	tumor necrosis factor-mediated signaling pathway	2	4	0.0212
GO:0009636	response to toxin	4	31	0.0214
GO:0002474	antigen processing and presentation of peptide antigen via MHC class I	3	15	0.0229

Table 4 continued on next page

GOBPID	Term	Count	Size	p-value
GO:0055088	lipid homeostasis	4	32	0.0237
GO:0010817	regulation of hormone levels	8	138	0.0270
GO:0042573	retinoic acid metabolic process	3	16	0.0270
GO:0006576	biogenic amine metabolic process	6	81	0.0282
GO:0060707	trophoblast giant cell differentiation	2	5	0.0326
GO:0000096	sulfur amino acid metabolic process	3	18	0.0373
GO:0042445	hormone metabolic process	6	87	0.0392
GO:0008299	isoprenoid biosynthetic process	3	19	0.0426
GO:0022600	digestive system process	3	19	0.0426
GO:0044092	negative regulation of molecular function	6	89	0.0427
GO:0042157	lipoprotein metabolic process	5	63	0.0437
GO:0006534	cysteine metabolic process	2	6	0.0437
GO:0006559	L-phenylalanine catabolic process	2	6	0.0437
GO:0042574	retinal metabolic process	2	6	0.0437
GO:0043288	apocarotenoid metabolic process	2	6	0.0437
GO:0043534	blood vessel endothelial cell migration	2	6	0.0437
GO:0009072	aromatic amino acid family metabolic process	3	20	0.0460
GO:0080134	regulation of response to stress	7	123	0.0486

Table 4: GO–Term enrichment for all genes showing a negative contribution ($\leq$ -2) in source 2. GOBPID shows the corresponding GO–Term ID. Term indicates the biological process. Count shows the number of genes found for this GO–Term and Size is the number of all genes for the corresponding GO–Term. The p-value is based on the conditional hypergeometric test, corrected by FDR.

Source 3

GOBPID	Term	Count	Size	p-value
GO:0007049	cell cycle	33	625	1.2149e-09
GO:0051301	cell division	20	261	4.6725e-08
GO:0022403	cell cycle phase	21	317	1.5188e-07
GO:0000279	M phase	19	279	4.5277e-07
GO:0000278	mitotic cell cycle	18	249	4.5277e-07
GO:0022402	cell cycle process	21	368	1.0151e-06
GO:0006260	DNA replication	13	127	1.0151e-06
GO:0000087	M phase of mitotic cell cycle	14	188	1.0849e-05
GO:0000280	nuclear division	14	188	1.0849e-05
GO:0007067	mitosis	14	188	1.0849e-05
GO:0048285	organelle fission	14	192	1.2824e-05
GO:0006270	DNA replication initiation	5	12	2.0454e-05
GO:0006261	DNA-dependent DNA replication	6	29	0.0001
GO:0050896	response to stimulus	45	1775	0.0001
GO:0006259	DNA metabolic process	17	377	0.0003
GO:0042221	response to chemical stimulus	20	543	0.0010
GO:0006268	DNA unwinding during replication	3	6	0.0024
GO:0032508	DNA duplex unwinding	3	6	0.0024
GO:0034641	cellular nitrogen compound metabolic process	11	204	0.0030
GO:0032392	DNA geometric change	3	7	0.0037
GO:0006575	cellular amino acid derivative metabolic process	8	129	0.0117
GO:0006275	regulation of DNA replication	3	12	0.0204
GO:0060192	negative regulation of lipase activity	2	3	0.0224
GO:0006519	cellular amino acid and derivative metabolic process	11	263	0.0224
GO:0009636	response to toxin	4	31	0.0263
GO:0009410	response to xenobiotic stimulus	3	15	0.0348
GO:0009987	cellular process	130	9162	0.0424
GO:0044270	nitrogen compound catabolic process	5	64	0.0499

Table 5: GO–Term enrichment for all genes showing a positive contribution (≥ 2) in source 3. GOBPID shows the corresponding GO–Term ID. Term indicates the biological process. Count shows the number of genes found for this GO–Term and Size is the number of all genes for the corresponding GO–Term. The p-value is based on the conditional hypergeometric test, corrected by FDR.

Source 4

GOBPID	Term	Count	Size	p-value
GO:0006412	translation	27	301	2.0126e-13
GO:0042989	sequestering of actin monomers	3	3	0.0006
GO:0044267	cellular protein metabolic process	46	1946	0.0006
GO:0051258	protein polymerization	8	72	0.0006
GO:0019538	protein metabolic process	50	2371	0.0038
GO:0019221	cytokine-mediated signaling pathway	6	53	0.0073

Table 6 continued on next page

GOBPID	Term	Count	Size	p-value
GO:0009611	response to wounding	13	313	0.0145
GO:0043623	cellular protein complex assembly	8	121	0.0145
GO:0008064	regulation of actin polymerization or depolymerization	5	45	0.0243
GO:0030832	regulation of actin filament length	5	46	0.0243
GO:0006950	response to stress	25	979	0.0248
GO:0032535	regulation of cellular component size	5	49	0.0262
GO:0006953	acute-phase response	4	28	0.0262
GO:0034621	cellular macromolecular complex subunit organization	10	222	0.0262
GO:0008154	actin polymerization or depolymerization	5	52	0.0262
GO:0032956	regulation of actin cytoskeleton organization	5	52	0.0262
GO:0006952	defense response	13	360	0.0262
GO:0032970	regulation of actin filament-based process	5	53	0.0265
GO:0034622	cellular macromolecular complex assembly	9	190	0.0281
GO:0043933	macromolecular complex subunit organization	11	278	0.0296
GO:0002474	antigen processing and presentation of peptide antigen via MHC class I	3	15	0.0374
GO:0042221	response to chemical stimulus	16	543	0.0415
GO:0033209	tumor necrosis factor-mediated signaling pathway	2	4	0.0416
GO:0006461	protein complex assembly	8	169	0.0444
GO:0070271	protein complex biogenesis	8	169	0.0444
GO:0007596	blood coagulation	5	66	0.0486
GO:0044087	regulation of cellular component biogenesis	5	66	0.0486
GO:0007599	hemostasis	5	67	0.0491
GO:0051235	maintenance of location	4	40	0.0491
GO:0030212	hyaluronan metabolic process	2	5	0.0491
GO:0050817	coagulation	5	69	0.0491
GO:0006414	translational elongation	3	19	0.0491
GO:0030833	regulation of actin filament polymerization	4	41	0.0491
GO:0010033	response to organic substance	10	270	0.0492
GO:0002449	lymphocyte mediated immunity	6	104	0.0492
GO:0065008	regulation of biological quality	23	989	0.0492
GO:0002526	acute inflammatory response	5	71	0.0492
GO:0006749	glutathione metabolic process	3	20	0.0494
GO:0050896	response to stimulus	35	1775	0.0494

Table 6: GO–Term enrichment for all genes showing a positive contribution (≥ 2) in source 4. GOBPID shows the corresponding GO–Term ID. Term indicates the biological process. Count shows the number of genes found for this GO–Term and Size is the number of all genes for the corresponding GO–Term. The p-value is based on the conditional hypergeometric test, corrected by FDR.

GOBPID	Term	Count	Size	p-value
GO:0006082	organic acid metabolic process	17	436	2.1201e-05
GO:0019752	carboxylic acid metabolic process	17	436	2.1201e-05
GO:0043436	oxoacid metabolic process	17	436	2.1201e-05
GO:0042180	cellular ketone metabolic process	17	447	2.2769e-05
GO:0006725	cellular aromatic compound metabolic process	8	98	0.0002

Table 7 continued on next page

GOBPID	Term	Count	Size	p-value
GO:0055114	oxidation reduction	16	572	0.0020
GO:0009308	amine metabolic process	11	283	0.0022
GO:0034641	cellular nitrogen compound metabolic process	9	204	0.0043
GO:0006519	cellular amino acid and derivative metabolic process	10	263	0.0051
GO:0009074	aromatic amino acid family catabolic process	3	11	0.0067
GO:0006629	lipid metabolic process	15	637	0.0146
GO:0032787	monocarboxylic acid metabolic process	9	253	0.0151
GO:0019439	aromatic compound catabolic process	3	16	0.0170
GO:0006520	cellular amino acid metabolic process	7	167	0.0233
GO:0044106	cellular amine metabolic process	7	167	0.0233
GO:0044255	cellular lipid metabolic process	13	547	0.0254
GO:0009072	aromatic amino acid family metabolic process	3	20	0.0257
GO:0009063	cellular amino acid catabolic process	4	47	0.0257
GO:0042537	benzene and derivative metabolic process	2	5	0.0263
GO:0006575	cellular amino acid derivative metabolic process	6	129	0.0263
GO:0006559	L-phenylalanine catabolic process	2	6	0.0371
GO:0009310	amine catabolic process	4	55	0.0382
GO:0042401	biogenic amine biosynthetic process	3	26	0.0417
GO:0050873	brown fat cell differentiation	3	27	0.0417
GO:0006558	L-phenylalanine metabolic process	2	7	0.0417
GO:0043255	regulation of carbohydrate biosynthetic process	2	7	0.0417
GO:0006954	inflammatory response	7	210	0.0497

Table 7: GO–Term enrichment for all genes showing a negative contribution ($\leq$ -2) in source 4. GOBPID shows the corresponding GO–Term ID. Term indicates the biological process. Count shows the number of genes found for this GO–Term and Size is the number of all genes for the corresponding GO–Term. The p-value is based on the conditional hypergeometric test, corrected by FDR.

Pathway enrichment

Source 1

KEGGID	Term	Count	Size	*p*-value
04630	Jak-STAT signaling pathway	7	134	0.0055
04115	p53 signaling pathway	5	63	0.0055
04610	Complement and coagulation cascades	5	65	0.0055
00072	Synthesis and degradation of ketone bodies	2	9	0.0475

Table 8: KEGG pathway enrichment for all genes showing a positive contribution ($\geq$ 2) in source 1. KEGGID shows the corresponding GO-Term ID. Term indicates the KEGG pathway. Count shows the number of genes found for this pathway and Size is the number of all genes for the corresponding pathway. The p-value is based on the conditional hypergeometric test, corrected by FDR.

KEGGID	Term	Count	Size	*p*-value
00010	Glycolysis / Gluconeogenesis	3	53	0.0349
01061	Biosynthesis of phenylpropanoids	3	56	0.0349
01064	Biosynthesis of alkaloids derived from ornithine, lysine and nicotinic acid	3	57	0.0349
01063	Biosynthesis of alkaloids derived from shikimate pathway	3	59	0.0349
01062	Biosynthesis of terpenoids and steroids	3	62	0.0349
04115	p53 signaling pathway	3	63	0.0349
01066	Biosynthesis of alkaloids derived from terpenoid and polyketide	3	67	0.0349
00710	Carbon fixation in photosynthetic organisms	2	21	0.0349
01065	Biosynthesis of alkaloids derived from histidine and purine	3	69	0.0349
00030	Pentose phosphate pathway	2	26	0.0476
01070	Biosynthesis of plant hormones	3	85	0.0489
00260	Glycine, serine and threonine metabolism	2	29	0.0489
05215	Prostate cancer	3	89	0.0489

Table 9: KEGG pathway enrichment for all genes showing a negative contribution ($\leq$ -2) in source 1. KEGGID shows the corresponding GO-Term ID. Term indicates the KEGG pathway. Count shows the number of genes found for this pathway and Size is the number of all genes for the corresponding pathway. The p-value is based on the conditional hypergeometric test, corrected by FDR.

Source 2

KEGGID	Term	Count	Size	*p*-value

Table 10: KEGG pathway enrichment for all genes showing a positive contribution ($\geq$ 2) in source 2. KEGGID shows the corresponding GO-Term ID. Term indicates the KEGG pathway. Count shows the number of genes found for this pathway and Size is the number of all genes for the corresponding pathway. The p-value is based on the conditional hypergeometric test, corrected by FDR.

KEGGID	Term	Count	Size	*p*-value
03010	Ribosome	36	70	3.6149e-46
00982	Drug metabolism - cytochrome P450	13	64	3.7474e-10
01100	Metabolic pathways	41	1011	7.5439e-08
00830	Retinol metabolism	10	51	7.7159e-08
00980	Metabolism of xenobiotics by cytochrome P450	10	58	2.3052e-07
04610	Complement and coagulation cascades	10	65	6.0271e-07
03320	PPAR signaling pathway	9	68	9.6594e-06
00040	Pentose and glucuronate interconversions	4	13	0.0003
00500	Starch and sucrose metabolism	5	32	0.0011
00072	Synthesis and degradation of ketone bodies	3	9	0.0026
00983	Drug metabolism - other enzymes	5	40	0.0027
00430	Taurine and hypotaurine metabolism	3	10	0.0031
00860	Porphyrin and chlorophyll metabolism	4	27	0.0051
00053	Ascorbate and aldarate metabolism	3	13	0.0058
00150	Androgen and estrogen metabolism	4	29	0.0058
00120	Primary bile acid biosynthesis	3	15	0.0085
00650	Butanoate metabolism	4	34	0.0095
01063	Biosynthesis of alkaloids derived from shikimate pathway	5	59	0.0103
00280	Valine, leucine and isoleucine degradation	4	45	0.0235
00232	Caffeine metabolism	2	7	0.0235

Table 11: KEGG pathway enrichment for all genes showing a negative contribution ($\leq$ -2) in source 2. KEGGID shows the corresponding GO-Term ID. Term indicates the KEGG pathway. Count shows the number of genes found for this pathway and Size is the number of all genes for the corresponding pathway. The p-value is based on the conditional hypergeometric test, corrected by FDR.

Source 3

KEGGID	Term	Count	Size	*p*-value
04110	Cell cycle	13	122	5.6684e-08
03030	DNA replication	7	35	3.1234e-06
03320	PPAR signaling pathway	6	68	0.0023
04115	p53 signaling pathway	5	63	0.0113
01100	Metabolic pathways	22	1011	0.0221
01040	Biosynthesis of unsaturated fatty acids	3	24	0.0241

table 13 continued on next page

KEGGID	Term	Count	Size	p-value
00480	Glutathione metabolism	4	51	0.0241
00980	Metabolism of xenobiotics by cytochrome P450	4	58	0.0337
00982	Drug metabolism - cytochrome P450	4	64	0.0427
00590	Arachidonic acid metabolism	4	68	0.0476

Table 12: KEGG pathway enrichment for all genes showing a positive contribution ($\geq$ 2) in source 3. KEGGID shows the corresponding GO-Term ID. Term indicates the KEGG pathway. Count shows the number of genes found for this pathway and Size is the number of all genes for the corresponding pathway. The p-value is based on the conditional hypergeometric test, corrected by FDR.

KEGGID	Term	Count	Size	p-value

Table 13: KEGG pathway enrichment for all genes showing a negative contribution ($\leq$ -2) in source 3. KEGGID shows the corresponding GO-Term ID. Term indicates the KEGG pathway. Count shows the number of genes found for this pathway and Size is the number of all genes for the corresponding pathway. The p-value is based on the conditional hypergeometric test, corrected by FDR.

Source 4

KEGGID	Term	Count	Size	p-value
03010	Ribosome	30	70	5.9938e-40
04610	Complement and coagulation cascades	7	65	0.0001

Table 14: KEGG pathway enrichment for all genes showing a positive contribution ($\geq$ 2) in source 4. KEGGID shows the corresponding GO-Term ID. Term indicates the KEGG pathway. Count shows the number of genes found for this pathway and Size is the number of all genes for the corresponding pathway. The p-value is based on the conditional hypergeometric test, corrected by FDR.

KEGGID	Term	Count	Size	p-value
01100	Metabolic pathways	23	1011	0.0003
01040	Biosynthesis of unsaturated fatty acids	4	24	0.0015
00980	Metabolism of xenobiotics by cytochrome P450	5	58	0.0028
00120	Primary bile acid biosynthesis	3	15	0.0046
00830	Retinol metabolism	4	51	0.0126
00982	Drug metabolism - cytochrome P450	4	64	0.0231
00590	Arachidonic acid metabolism	4	68	0.0231

table 15 continued on next page

KEGGID	Term	Count	Size	p-value
03320	PPAR signaling pathway	4	68	0.0231
00430	Taurine and hypotaurine metabolism	2	10	0.0265

Table 15: KEGG pathway enrichment for all genes showing a negative contribution ($\leq$ -2) in source 4. KEGGID shows the corresponding GO-Term ID. Term indicates the KEGG pathway. Count shows the number of genes found for this pathway and Size is the number of all genes for the corresponding pathway. The p-value is based on the conditional hypergeometric test, corrected by FDR.

PCA results

To validated our findings obtained by GraDE we first used PCA, which is a standard technique to explore microarray data. We applied PCA to the same set of expressed gene compared to GraDe. We inferred four component similar to GraDe and define for each component two submodes by grouping genes with a threshold $\geq +2$ sigma and a second set of genes having a component weight of ≤ -2 sigma.

The following subsections list the significantly enriched GO–terms and pathways found in the source matrix obtained by PCA.

Eigenvalues of the PCA

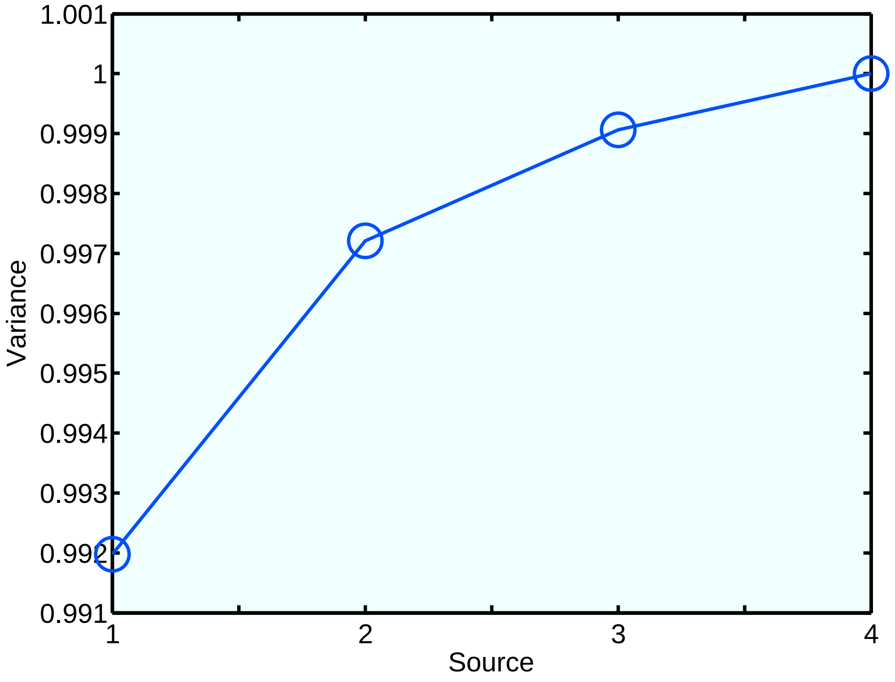

Additional Figure 1: We applied the PCA to the *Il-6* stimulated primary mouse hepatocytes. The figure shows the cumulative eigenvalues of the PCA for the 4 different sources. The result indicates that source 1 contains 99% of the variance in the time-course data.

GO-Term enrichment

Source 1

GOBPID	Term	Count	Size	p-value

Table 16: GO -Term enrichment for all genes showing a positive contribution (≥ 2) in source 1. GOBPID shows the corresponding GO-Term ID. Term indicates the biological process. Count shows the number of genes found for this GO-Term and Size is the number of all genes for the corresponding GO-Term. The p-value is based on the conditional hypergeometric test, corrected by FDR.

GOBPID	Term	Count	Size	p-value
GO:0006412	translation	49	301	1.5790e-30
GO:0044267	cellular protein metabolic process	78	1946	1.4316e-10
GO:0019538	protein metabolic process	88	2371	1.4316e-10
GO:0065008	regulation of biological quality	39	989	0.0006
GO:0007596	blood coagulation	9	66	0.0006
GO:0007599	hemostasis	9	67	0.0006
GO:0009611	response to wounding	19	313	0.0006
GO:0050817	coagulation	9	69	0.0006
GO:0042060	wound healing	10	94	0.0010
GO:0008152	metabolic process	147	6575	0.0018
GO:0006414	translational elongation	5	19	0.0022
GO:0050878	regulation of body fluid levels	9	86	0.0026
GO:0006091	generation of precursor metabolites and energy	15	240	0.0028
GO:0009605	response to external stimulus	23	516	0.0049
GO:0043243	positive regulation of protein complex disassembly	3	5	0.0054
GO:0006950	response to stress	34	979	0.0108
GO:0006457	protein folding	9	111	0.0133
GO:0042572	retinol metabolic process	3	7	0.0133
GO:0044237	cellular metabolic process	126	5627	0.0133
GO:0006119	oxidative phosphorylation	6	48	0.0133
GO:0006818	hydrogen transport	6	48	0.0133
GO:0015992	proton transport	6	48	0.0133
GO:0050896	response to stimulus	51	1775	0.0145
GO:0002526	acute inflammatory response	7	71	0.0145
GO:0006766	vitamin metabolic process	7	71	0.0145
GO:0042221	response to chemical stimulus	22	543	0.0145
GO:0015985	energy coupled proton transport, down electrochemical gradient	5	33	0.0145
GO:0015986	ATP synthesis coupled proton transport	5	33	0.0145
GO:0006869	lipid transport	8	96	0.0158
GO:0044238	primary metabolic process	128	5820	0.0181
GO:0009201	ribonucleoside triphosphate biosynthetic process	7	76	0.0181
GO:0009206	purine ribonucleoside triphosphate biosynthetic process	7	76	0.0181
GO:0034220	ion transmembrane transport	5	36	0.0181
GO:0006979	response to oxidative stress	6	55	0.0181
GO:0009145	purine nucleoside triphosphate biosynthetic process	7	77	0.0181
GO:0033344	cholesterol efflux	3	9	0.0181
GO:0043933	macromolecular complex subunit organization	14	278	0.0183
GO:0009142	nucleoside triphosphate biosynthetic process	7	78	0.0183
GO:0051130	positive regulation of cellular component organization	7	79	0.0193
GO:0009205	purine ribonucleoside triphosphate metabolic process	7	80	0.0195
GO:0055114	oxidation reduction	22	573	0.0195
GO:0000302	response to reactive oxygen species	4	22	0.0195
GO:0009199	ribonucleoside triphosphate metabolic process	7	81	0.0195
GO:0044249	cellular biosynthetic process	78	3167	0.0195
GO:0034614	cellular response to reactive oxygen species	3	10	0.0195

table 17 continued on next page

GOBPID	Term	Count	Size	p-value
GO:0042744	hydrogen peroxide catabolic process	3	10	0.0195
GO:0070301	cellular response to hydrogen peroxide	3	10	0.0195
GO:0002682	regulation of immune system process	13	258	0.0224
GO:0009144	purine nucleoside triphosphate metabolic process	7	85	0.0233
GO:0034097	response to cytokine stimulus	4	24	0.0233
GO:0009058	biosynthetic process	79	3244	0.0233
GO:0019852	L-ascorbic acid metabolic process	2	3	0.0261
GO:0030836	positive regulation of actin filament depolymerization	2	3	0.0261
GO:0042989	sequestering of actin monomers	2	3	0.0261
GO:0009141	nucleoside triphosphate metabolic process	7	89	0.0281
GO:0002541	activation of plasma proteins involved in acute inflammatory response	4	26	0.0283
GO:0006956	complement activation	4	26	0.0283
GO:0009152	purine ribonucleotide biosynthetic process	7	90	0.0284
GO:0008064	regulation of actin polymerization or depolymerization	5	45	0.0298
GO:0006959	humoral immune response	5	46	0.0318
GO:0030832	regulation of actin filament length	5	46	0.0318
GO:0006754	ATP biosynthetic process	6	69	0.0331
GO:0034599	cellular response to oxidative stress	3	13	0.0331
GO:0009260	ribonucleotide biosynthetic process	7	94	0.0331
GO:0006775	fat-soluble vitamin metabolic process	4	28	0.0331
GO:0048583	regulation of response to stimulus	12	247	0.0349
GO:0019725	cellular homeostasis	13	281	0.0349
GO:0002455	humoral immune response mediated by circulating immunoglobulin	4	29	0.0362
GO:0032535	regulation of cellular component size	5	49	0.0369
GO:0008203	cholesterol metabolic process	6	72	0.0369
GO:0051258	protein polymerization	6	72	0.0369
GO:0009150	purine ribonucleotide metabolic process	7	98	0.0377
GO:0033209	tumor necrosis factor-mediated signaling pathway	2	4	0.0380
GO:0046034	ATP metabolic process	6	73	0.0380
GO:0034621	cellular macromolecular complex subunit organization	11	222	0.0417
GO:0006575	cellular amino acid derivative metabolic process	8	129	0.0420
GO:0051493	regulation of cytoskeleton organization	6	75	0.0420
GO:0008154	actin polymerization or depolymerization	5	52	0.0428
GO:0032956	regulation of actin cytoskeleton organization	5	52	0.0428
GO:0032970	regulation of actin filament-based process	5	53	0.0460
GO:0009259	ribonucleotide metabolic process	7	104	0.0470
GO:0006081	cellular aldehyde metabolic process	3	16	0.0471
GO:0042542	response to hydrogen peroxide	3	16	0.0471
GO:0050818	regulation of coagulation	3	16	0.0471
GO:0010638	positive regulation of organelle organization	4	33	0.0473
GO:0016125	sterol metabolic process	6	79	0.0488
GO:0045087	innate immune response	7	106	0.0488
GO:0045454	cell redox homeostasis	5	55	0.0493

Table 17: GO -Term enrichment for all genes showing a negative contribution ($\leq$ -2) in source 1. GOBPID shows the corresponding GO-Term ID. Term indicates the biological process. Count shows the number of genes found for this GO-Term and Size is the number of all genes for the corresponding GO-Term. The p-value is based on the conditional hypergeometric test, corrected by FDR.

Source 2

GOBPID	Term	Count	Size	p-value
GO:0055114	oxidation reduction	25	573	2.0766e-08
GO:0006082	organic acid metabolic process	17	436	5.9820e-05
GO:0019752	carboxylic acid metabolic process	17	436	5.9820e-05
GO:0043436	oxoacid metabolic process	17	436	5.9820e-05
GO:0042180	cellular ketone metabolic process	17	447	6.7977e-05
GO:0045444	fat cell differentiation	6	63	0.0026
GO:0009093	cysteine catabolic process	2	2	0.0060
GO:0019448	L-cysteine catabolic process	2	2	0.0060
GO:0019452	L-cysteine catabolic process to taurine	2	2	0.0060
GO:0046439	L-cysteine metabolic process	2	2	0.0060
GO:0032787	monocarboxylic acid metabolic process	10	253	0.0060
GO:0050873	brown fat cell differentiation	4	27	0.0060
GO:0009310	amine catabolic process	5	55	0.0078
GO:0006575	cellular amino acid derivative metabolic process	7	129	0.0087
GO:0009308	amine metabolic process	10	283	0.0110
GO:0000098	sulfur amino acid catabolic process	2	3	0.0110
GO:0044273	sulfur compound catabolic process	2	3	0.0110
GO:0044270	nitrogen compound catabolic process	5	64	0.0115
GO:0019530	taurine metabolic process	2	4	0.0192
GO:0034641	cellular nitrogen compound metabolic process	8	204	0.0192
GO:0000096	sulfur amino acid metabolic process	3	18	0.0194
GO:0006519	cellular amino acid and derivative metabolic process	9	263	0.0209
GO:0009063	cellular amino acid catabolic process	4	47	0.0278
GO:0006631	fatty acid metabolic process	7	175	0.0324
GO:0006534	cysteine metabolic process	2	6	0.0335
GO:0050872	white fat cell differentiation	2	6	0.0335
GO:0060710	chorio-allantoic fusion	2	6	0.0335
GO:0044255	cellular lipid metabolic process	13	547	0.0335

Table 18: GO -Term enrichment for all genes showing a positive contribution (≥ 2) in source 2. GOBPID shows the corresponding GO-Term ID. Term indicates the biological process. Count shows the number of genes found for this GO-Term and Size is the number of all genes for the corresponding GO-Term. The p-value is based on the conditional hypergeometric test, corrected by FDR.

GOBPID	Term	Count	Size	p-value
GO:0051301	cell division	20	261	5.6682e-09
GO:0007049	cell cycle	29	625	8.9637e-09
GO:0000279	M phase	18	279	2.2417e-07
GO:0022403	cell cycle phase	19	317	2.2417e-07
GO:0000278	mitotic cell cycle	17	249	2.2417e-07
GO:0000087	M phase of mitotic cell cycle	15	188	2.2417e-07
GO:0000280	nuclear division	15	188	2.2417e-07
GO:0007067	mitosis	15	188	2.2417e-07
GO:0048285	organelle fission	15	192	2.6676e-07

table 19 continued on next page

GOBPID	Term	Count	Size	p-value
GO:0022402	cell cycle process	19	368	1.5842e-06
GO:0050896	response to stimulus	39	1775	0.0006
GO:0006950	response to stress	26	979	0.0013
GO:0009611	response to wounding	12	313	0.0121
GO:0030261	chromosome condensation	3	15	0.0394

Table 19: GO -Term enrichment for all genes showing a negative contribution ($\leq$ -2) in source 2. GOBPID shows the corresponding GO-Term ID. Term indicates the biological process. Count shows the number of genes found for this GO-Term and Size is the number of all genes for the corresponding GO-Term. The p-value is based on the conditional hypergeometric test, corrected by FDR.

Source 3

GOBPID	Term	Count	Size	p-value
GO:0006082	organic acid metabolic process	23	436	5.0906e-07
GO:0019752	carboxylic acid metabolic process	23	436	5.0906e-07
GO:0043436	oxoacid metabolic process	23	436	5.0906e-07
GO:0042180	cellular ketone metabolic process	23	447	6.1701e-07
GO:0006629	lipid metabolic process	27	637	1.1627e-06
GO:0044255	cellular lipid metabolic process	24	547	4.0161e-06
GO:0008202	steroid metabolic process	13	155	5.1071e-06
GO:0048008	platelet-derived growth factor receptor signaling pathway	5	18	0.0002
GO:0055114	oxidation reduction	21	573	0.0004
GO:0034641	cellular nitrogen compound metabolic process	12	204	0.0005
GO:0032787	monocarboxylic acid metabolic process	13	253	0.0008
GO:0060324	face development	4	15	0.0019
GO:0008152	metabolic process	101	6575	0.0031
GO:0006631	fatty acid metabolic process	10	175	0.0031
GO:0060322	head development	4	18	0.0034
GO:0009093	cysteine catabolic process	2	2	0.0074
GO:0019448	L-cysteine catabolic process	2	2	0.0074
GO:0019452	L-cysteine catabolic process to taurine	2	2	0.0074
GO:0046439	L-cysteine metabolic process	2	2	0.0074
GO:0016054	organic acid catabolic process	6	69	0.0078
GO:0046395	carboxylic acid catabolic process	6	69	0.0078
GO:0008203	cholesterol metabolic process	6	72	0.0094
GO:0009063	cellular amino acid catabolic process	5	47	0.0098
GO:0009074	aromatic amino acid family catabolic process	3	11	0.0101
GO:0060325	face morphogenesis	3	11	0.0101
GO:0006519	cellular amino acid and derivative metabolic process	11	263	0.0111
GO:0006694	steroid biosynthetic process	6	77	0.0111
GO:0050896	response to stimulus	37	1775	0.0111
GO:0008206	bile acid metabolic process	3	12	0.0115
GO:0016125	sterol metabolic process	6	79	0.0115

table 20 continued on next page

GOBPID	Term	Count	Size	p-value
GO:0000098	sulfur amino acid catabolic process	2	3	0.0127
GO:0044273	sulfur compound catabolic process	2	3	0.0127
GO:0060192	negative regulation of lipase activity	2	3	0.0127
GO:0060323	head morphogenesis	3	13	0.0127
GO:0009310	amine catabolic process	5	55	0.0136
GO:0009308	amine metabolic process	11	283	0.0152
GO:0060021	palate development	4	33	0.0162
GO:0019439	aromatic compound catabolic process	3	16	0.0205
GO:0019530	taurine metabolic process	2	4	0.0205
GO:0006520	cellular amino acid metabolic process	8	167	0.0205
GO:0044106	cellular amine metabolic process	8	167	0.0205
GO:0006575	cellular amino acid derivative metabolic process	7	129	0.0205
GO:0044271	nitrogen compound biosynthetic process	7	130	0.0209
GO:0044270	nitrogen compound catabolic process	5	64	0.0218
GO:0000096	sulfur amino acid metabolic process	3	18	0.0263
GO:0008610	lipid biosynthetic process	10	268	0.0273
GO:0006569	tryptophan catabolic process	2	5	0.0273
GO:0010761	fibroblast migration	2	5	0.0273
GO:0042436	indole derivative catabolic process	2	5	0.0273
GO:0046218	indolalkylamine catabolic process	2	5	0.0273
GO:0010171	body morphogenesis	3	19	0.0273
GO:0006066	alcohol metabolic process	11	320	0.0287
GO:0009072	aromatic amino acid family metabolic process	3	20	0.0306
GO:0006534	cysteine metabolic process	2	6	0.0381

Table 20: GO -Term enrichment for all genes showing a positive contribution (≥ 2) in source 3. GOBPID shows the corresponding GO-Term ID. Term indicates the biological process. Count shows the number of genes found for this GO-Term and Size is the number of all genes for the corresponding GO-Term. The p-value is based on the conditional hypergeometric test, corrected by FDR.

GOBPID	Term	Count	Size	p-value
GO:0006002	fructose 6-phosphate metabolic process	2	2	0.0151
GO:0051239	regulation of multicellular organismal process	11	640	0.0499
GO:0006000	fructose metabolic process	2	5	0.0499

Table 21: GO -Term enrichment for all genes showing a negative contribution ($\leq$ -2) in source 3. GOBPID shows the corresponding GO-Term ID. Term indicates the biological process. Count shows the number of genes found for this GO-Term and Size is the number of all genes for the corresponding GO-Term. The p-value is based on the conditional hypergeometric test, corrected by FDR.

Source 4

GOBPID	Term	Count	Size	p-value
GO:0002526	acute inflammatory response	6	71	0.0377
GO:0009611	response to wounding	11	313	0.0460
GO:0040007	growth	11	323	0.0460

Table 22: GO -Term enrichment for all genes showing a positive contribution ($\geq$ 2) in source 4. GOBPID shows the corresponding GO-Term ID. Term indicates the biological process. Count shows the number of genes found for this GO-Term and Size is the number of all genes for the corresponding GO-Term. The p-value is based on the conditional hypergeometric test, corrected by FDR.

GOBPID	Term	Count	Size	p-value
GO:0007049	cell cycle	21	625	0.0016
GO:0051301	cell division	13	261	0.0016
GO:0000278	mitotic cell cycle	12	249	0.0037
GO:0055114	oxidation reduction	17	573	0.0206
GO:0022403	cell cycle phase	12	317	0.0206
GO:0022402	cell cycle process	13	368	0.0206
GO:0006270	DNA replication initiation	3	12	0.0342
GO:0006260	DNA replication	7	127	0.0471

Table 23: GO -Term enrichment for all genes showing a negative contribution ($\leq$ -2) in source 4. GOBPID shows the corresponding GO-Term ID. Term indicates the biological process. Count shows the number of genes found for this GO-Term and Size is the number of all genes for the corresponding GO-Term. The p-value is based on the conditional hypergeometric test, corrected by FDR.

Pathway enrichment

Source 1

KEGGID	Term	Count	Size	*p*-value

Table 24: KEGG pathway enrichment for all genes showing a positive contribution (≥ 2) in source 1. KEGGID shows the corresponding GO-Term ID. Term indicates the KEGG pathway. Count shows the number of genes found for this pathway and Size is the number of all genes for the corresponding pathway. The p-value is based on the conditional hypergeometric test, corrected by FDR.

KEGGID	Term	Count	Size	*p*-value
03010	Ribosome	58	70	6.6278e-89
00190	Oxidative phosphorylation	18	108	6.8392e-11
05012	Parkinson's disease	17	103	2.3039e-10
05016	Huntington's disease	20	156	2.8967e-10
05010	Alzheimer's disease	16	152	5.0518e-07
04610	Complement and coagulation cascades	11	65	5.0518e-07
04612	Antigen processing and presentation	9	66	5.3586e-05
00980	Metabolism of xenobiotics by cytochrome P450	8	58	0.0001
01100	Metabolic pathways	36	1011	0.0015
00982	Drug metabolism - cytochrome P450	7	64	0.0019
04260	Cardiac muscle contraction	7	66	0.0021
00480	Glutathione metabolism	6	51	0.0032
00830	Retinol metabolism	5	51	0.0212
00010	Glycolysis / Gluconeogenesis	5	53	0.0234
01061	Biosynthesis of phenylpropanoids	5	56	0.0277
00710	Carbon fixation in photosynthetic organisms	3	21	0.0442
00680	Methane metabolism	2	7	0.0442

Table 25: KEGG pathway enrichment for all genes showing a negative contribution ($\leq$ -2) in source 1. KEGGID shows the corresponding GO-Term ID. Term indicates the KEGG pathway. Count shows the number of genes found for this pathway and Size is the number of all genes for the corresponding pathway. The p-value is based on the conditional hypergeometric test, corrected by FDR.

Source 2

KEGGID	Term	Count	Size	*p*-value
00982	Drug metabolism - cytochrome P450	10	64	1.2665e-08
01100	Metabolic pathways	24	1011	0.0001
00830	Retinol metabolism	6	51	0.0001
00980	Metabolism of xenobiotics by cytochrome P450	6	58	0.0001

table 27 continued on next page

KEGGID	Term	Count	Size	p-value
01063	Biosynthesis of alkaloids derived from shikimate pathway	6	59	0.0001
00430	Taurine and hypotaurine metabolism	3	10	0.0010
00051	Fructose and mannose metabolism	4	37	0.0033
00983	Drug metabolism - other enzymes	4	40	0.0040
01040	Biosynthesis of unsaturated fatty acids	3	24	0.0105
00232	Caffeine metabolism	2	7	0.0130
00260	Glycine, serine and threonine metabolism	3	29	0.0151
04610	Complement and coagulation cascades	4	65	0.0168
00561	Glycerolipid metabolism	3	45	0.0445
00120	Primary bile acid biosynthesis	2	15	0.0445

Table 26: KEGG pathway enrichment for all genes showing a positive contribution (≥ 2) in source 2. KEGGID shows the corresponding GO-Term ID. Term indicates the KEGG pathway. Count shows the number of genes found for this pathway and Size is the number of all genes for the corresponding pathway. The p-value is based on the conditional hypergeometric test, corrected by FDR.

KEGGID	Term	Count	Size	p-value
04110	Cell cycle	10	122	3.3219e-05
04630	Jak-STAT signaling pathway	9	134	0.0003
04115	p53 signaling pathway	6	63	0.0010

Table 27: KEGG pathway enrichment for all genes showing a negative contribution ($\leq$ -2) in source 2. KEGGID shows the corresponding GO-Term ID. Term indicates the KEGG pathway. Count shows the number of genes found for this pathway and Size is the number of all genes for the corresponding pathway. The p-value is based on the conditional hypergeometric test, corrected by FDR.

Source 3

KEGGID	Term	Count	Size	p-value
01100	Metabolic pathways	35	1011	9.2802e-08
03320	PPAR signaling pathway	9	68	2.6865e-06
00120	Primary bile acid biosynthesis	4	15	0.0005
00982	Drug metabolism - cytochrome P450	6	64	0.0017
00430	Taurine and hypotaurine metabolism	3	10	0.0029
00830	Retinol metabolism	5	51	0.0039
01040	Biosynthesis of unsaturated fatty acids	3	24	0.0319
00980	Metabolism of xenobiotics by cytochrome P450	4	58	0.0457
00072	Synthesis and degradation of ketone bodies	2	9	0.0457

Table 28: KEGG pathway enrichment for all genes showing a positive contribution ($\geq$ 2) in source 3. KEGGID shows the corresponding GO-Term ID. Term indicates the KEGG pathway. Count shows the number of genes found for this pathway and Size is the number of all genes for the corresponding pathway. The p-value is based on the conditional hypergeometric test, corrected by FDR.

KEGGID	Term	Count	Size	p-value
05215	Prostate cancer	4	89	0.0436

Table 29: KEGG pathway enrichment for all genes showing a negative contribution ($\leq$ -2) in source 3. KEGGID shows the corresponding GO-Term ID. Term indicates the KEGG pathway. Count shows the number of genes found for this pathway and Size is the number of all genes for the corresponding pathway. The p-value is based on the conditional hypergeometric test, corrected by FDR.

Source 4

KEGGID	Term	Count	Size	p-value
04610	Complement and coagulation cascades	7	65	6.0750e-05

Table 30: KEGG pathway enrichment for all genes showing a positive contribution ($\geq$ 2) in source 4. KEGGID shows the corresponding GO-Term ID. Term indicates the KEGG pathway. Count shows the number of genes found for this pathway and Size is the number of all genes for the corresponding pathway. The p-value is based on the conditional hypergeometric test, corrected by FDR.

KEGGID	Term	Count	Size	p-value
04115	p53 signaling pathway	6	63	0.0021
04110	Cell cycle	7	122	0.0058

Table 31: KEGG pathway enrichment for all genes showing a negative contribution ($\leq$ -2) in source 4. KEGGID shows the corresponding GO-Term ID. Term indicates the KEGG pathway. Count shows the number of genes found for this pathway and Size is the number of all genes for the corresponding pathway. The p-value is based on the conditional hypergeometric test, corrected by FDR.

Result of the k-means clustering

To provide an objective comparison of k-means clustering with GraDe and PCA, we first applied a feature selection step to ensure that all methods selected an approximately equal number of genes, as proposed in Teschendorff et al. 2007. We ranked all expressed genes accordingly to their expression variance across the time-course and then selected the top 15% variable genes. Having the selected features, clustering was then performed using a robust version of k-means clustering, where k was set to 8 in order to match the same number of submodes inferred by GraDe and PCA.

GO-Term enrichment

GOBPID	Term	Count	Size	p-value

Table 32: GO -Term enrichment for all genes in cluster 1. GOBPID shows the corresponding GO-Term ID. Term indicates the biological process. Count shows the number of genes found for this GO-Term and Size is the number of all genes for the corresponding GO-Term. The p-value is based on the conditional hypergeometric test, corrected by FDR.

GOBPID	Term	Count	Size	p-value
GO:0034504	protein localization in nucleus	4	68	0.0252
GO:0033365	protein localization in organelle	4	85	0.0252
GO:0048147	negative regulation of fibroblast proliferation	2	7	0.0252
GO:0006996	organelle organization	10	935	0.0252
GO:0032271	regulation of protein polymerization	3	45	0.0252
GO:0043254	regulation of protein complex assembly	3	45	0.0252
GO:0007275	multicellular organismal development	16	2280	0.0252
GO:0033043	regulation of organelle organization	4	114	0.0252
GO:0048523	negative regulation of cellular process	10	975	0.0252
GO:0048608	reproductive structure development	4	118	0.0252
GO:0016568	chromatin modification	5	215	0.0252
GO:0043623	cellular protein complex assembly	4	121	0.0252
GO:0048856	anatomical structure development	14	1957	0.0338
GO:0048519	negative regulation of biological process	10	1083	0.0338
GO:0006606	protein import into nucleus	3	64	0.0338
GO:0051170	nuclear import	3	64	0.0338
GO:0044087	regulation of cellular component biogenesis	3	66	0.0338
GO:0000060	protein import into nucleus, translocation	2	16	0.0338
GO:0006325	chromatin organization	5	264	0.0338
GO:0003006	reproductive developmental process	4	152	0.0338

table 33 continued on next page

GOBPID	Term	Count	Size	p-value
GO:0048144	fibroblast proliferation	2	17	0.0338
GO:0048145	regulation of fibroblast proliferation	2	17	0.0338
GO:0008406	gonad development	3	70	0.0338
GO:0032268	regulation of cellular protein metabolic process	5	272	0.0338
GO:0051258	protein polymerization	3	72	0.0343
GO:0007162	negative regulation of cell adhesion	2	18	0.0343
GO:0051493	regulation of cytoskeleton organization	3	75	0.0356
GO:0006461	protein complex assembly	4	169	0.0356
GO:0070271	protein complex biogenesis	4	169	0.0356
GO:0048731	system development	13	1841	0.0356
GO:0008285	negative regulation of cell proliferation	4	171	0.0356
GO:0017038	protein import	3	80	0.0369
GO:0045185	maintenance of protein location	2	22	0.0381
GO:0045137	development of primary sexual characteristics	3	84	0.0381
GO:0030837	negative regulation of actin filament polymerization	2	23	0.0381
GO:0032502	developmental process	16	2680	0.0381
GO:0032269	negative regulation of cellular protein metabolic process	3	88	0.0381
GO:0016043	cellular component organization	12	1673	0.0381
GO:0007569	cell aging	2	24	0.0381
GO:0031333	negative regulation of protein complex assembly	2	24	0.0381
GO:0032272	negative regulation of protein polymerization	2	24	0.0381
GO:0034622	cellular macromolecular complex assembly	4	190	0.0381
GO:0034613	cellular protein localization	5	319	0.0381
GO:0070727	cellular macromolecule localization	5	321	0.0382
GO:0040007	growth	5	323	0.0383
GO:0051248	negative regulation of protein metabolic process	3	92	0.0383
GO:0051246	regulation of protein metabolic process	5	326	0.0383
GO:0051276	chromosome organization	5	330	0.0387
GO:0001544	initiation of primordial ovarian follicle growth	1	1	0.0387
GO:0042149	cellular response to glucose starvation	1	1	0.0387
GO:0046370	fructose biosynthetic process	1	1	0.0387
GO:0051154	negative regulation of striated muscle cell differentiation	1	1	0.0387
GO:0060431	primary lung bud formation	1	1	0.0387
GO:0060449	bud elongation involved in lung branching	1	1	0.0387
GO:0006913	nucleocytoplasmic transport	3	99	0.0394
GO:0051169	nuclear transport	3	100	0.0399
GO:0007548	sex differentiation	3	105	0.0449
GO:0034621	cellular macromolecular complex subunit organization	4	222	0.0490

Table 33: GO -Term enrichment for all genes in cluster 2. GOBPID shows the corresponding GO-Term ID. Term indicates the biological process. Count shows the number of genes found for this GO-Term and Size is the number of all genes for the corresponding GO-Term. The p-value is based on the conditional hypergeometric test, corrected by FDR.

GOBPID	Term	Count	Size	p-value
GO:0050896	response to stimulus	24	1775	0.0013

table 34 continued on next page

GOBPID	Term	Count	Size	p-value
GO:0000278	mitotic cell cycle	9	249	0.0013
GO:0006950	response to stress	17	979	0.0013
GO:0051301	cell division	9	261	0.0013
GO:0007049	cell cycle	13	625	0.0023
GO:0009611	response to wounding	9	313	0.0036
GO:0022403	cell cycle phase	9	317	0.0036
GO:0000087	M phase of mitotic cell cycle	7	188	0.0037
GO:0000280	nuclear division	7	188	0.0037
GO:0007067	mitosis	7	188	0.0037
GO:0048285	organelle fission	7	192	0.0038
GO:0009605	response to external stimulus	11	516	0.0038
GO:0000279	M phase	8	279	0.0051
GO:0022402	cell cycle process	9	368	0.0058
GO:0043066	negative regulation of apoptosis	6	202	0.0288
GO:0043069	negative regulation of programmed cell death	6	206	0.0288
GO:0060548	negative regulation of cell death	6	206	0.0288
GO:0007059	chromosome segregation	3	41	0.0486

Table 34: GO -Term enrichment for all genes in cluster 3. GOBPID shows the corresponding GO-Term ID. Term indicates the biological process. Count shows the number of genes found for this GO-Term and Size is the number of all genes for the corresponding GO-Term. The p-value is based on the conditional hypergeometric test, corrected by FDR.

GOBPID	Term	Count	Size	p-value
GO:0050832	defense response to fungus	1	3	0.0242
GO:0060708	spongiotrophoblast differentiation	1	3	0.0242
GO:0060707	trophoblast giant cell differentiation	1	5	0.0242
GO:0046627	negative regulation of insulin receptor signaling pathway	1	6	0.0242
GO:0046626	regulation of insulin receptor signaling pathway	1	7	0.0242
GO:0060670	branching involved in embryonic placenta morphogenesis	1	8	0.0242
GO:0060712	spongiotrophoblast layer development	1	8	0.0242
GO:0060669	embryonic placenta morphogenesis	1	10	0.0242
GO:0060706	cell differentiation involved in embryonic placenta development	1	10	0.0242
GO:0009620	response to fungus	1	12	0.0261
GO:0060713	labyrinthine layer morphogenesis	1	16	0.0309
GO:0060674	placenta blood vessel development	1	17	0.0309
GO:0006879	cellular iron ion homeostasis	1	21	0.0352
GO:0008286	insulin receptor signaling pathway	1	27	0.0392
GO:0055072	iron ion homeostasis	1	27	0.0392
GO:0060711	labyrinthine layer development	1	33	0.0449

Table 35: GO -Term enrichment for all genes in cluster 4. GOBPID shows the corresponding GO-Term ID. Term indicates the biological process. Count shows the number of genes found for this GO-Term and Size is the number of all genes for the corresponding GO-Term. The p-value is based on the conditional hypergeometric test, corrected by FDR.

GOBPID	Term	Count	Size	p-value
GO:0044255	cellular lipid metabolic process	14	547	0.0005
GO:0006629	lipid metabolic process	15	637	0.0005
GO:0006082	organic acid metabolic process	12	436	0.0006
GO:0019752	carboxylic acid metabolic process	12	436	0.0006
GO:0043436	oxoacid metabolic process	12	436	0.0006
GO:0042180	cellular ketone metabolic process	12	447	0.0006
GO:0032787	monocarboxylic acid metabolic process	9	253	0.0010
GO:0008202	steroid metabolic process	7	155	0.0020
GO:0042537	benzene and derivative metabolic process	2	5	0.0230
GO:0006631	fatty acid metabolic process	6	175	0.0288
GO:0009636	response to toxin	3	31	0.0391
GO:0008203	cholesterol metabolic process	4	72	0.0391

Table 36: GO -Term enrichment for all genes in cluster 5. GOBPID shows the corresponding GO-Term ID. Term indicates the biological process. Count shows the number of genes found for this GO-Term and Size is the number of all genes for the corresponding GO-Term. The p-value is based on the conditional hypergeometric test, corrected by FDR.

GOBPID	Term	Count	Size	p-value
GO:0055114	oxidation reduction	16	572	0.0083
GO:0030193	regulation of blood coagulation	3	10	0.0209
GO:0006066	alcohol metabolic process	10	319	0.0455
GO:0050818	regulation of coagulation	3	16	0.0455
GO:0045682	regulation of epidermis development	3	17	0.0455
GO:0042730	fibrinolysis	2	4	0.0456

Table 37: GO -Term enrichment for all genes in cluster 6. GOBPID shows the corresponding GO-Term ID. Term indicates the biological process. Count shows the number of genes found for this GO-Term and Size is the number of all genes for the corresponding GO-Term. The p-value is based on the conditional hypergeometric test, corrected by FDR.

GOBPID	Term	Count	Size	p-value

Table 38: GO -Term enrichment for all genes in cluster 7. GOBPID shows the corresponding GO-Term ID. Term indicates the biological process. Count shows the number of genes found for this GO-Term and Size is the number of all genes for the corresponding GO-Term. The p-value is based on the conditional hypergeometric test, corrected by FDR.

GOBPID	Term	Count	Size	p-value
GO:0006260	DNA replication	9	127	0.0410
GO:0006807	nitrogen compound metabolic process	67	3201	0.0410
GO:0043618	regulation of transcription from RNA polymerase II promoter in response to stress	2	2	0.0410
GO:0043619	regulation of transcription from RNA polymerase II promoter in response to oxidative stress	2	2	0.0410
GO:0043620	regulation of transcription in response to stress	2	2	0.0410
GO:0006139	nucleobase, nucleoside, nucleotide and nucleic acid metabolic process	61	2918	0.0410

Table 39: GO -Term enrichment for all genes in cluster 8. GOBPID shows the corresponding GO-Term ID. Term indicates the biological process. Count shows the number of genes found for this GO-Term and Size is the number of all genes for the corresponding GO-Term. The p-value is based on the conditional hypergeometric test, corrected by FDR.

Pathway enrichment

KEGGID	Term	Count	Size	*p*-value

Table 40: KEGG pathway enrichment for all genes in cluster 1. KEGGID shows the corresponding GO-Term ID. Term indicates the KEGG pathway. Count shows the number of genes found for this pathway and Size is the number of all genes for the corresponding pathway. The p-value is based on the conditional hypergeometric test, corrected by FDR.

KEGGID	Term	Count	Size	*p*-value
05220	Chronic myeloid leukemia	4	74	0.0020
04722	Neurotrophin signaling pathway	4	124	0.0046
05213	Endometrial cancer	3	51	0.0046
05223	Non-small cell lung cancer	3	54	0.0046
04115	p53 signaling pathway	3	63	0.0058
04012	ErbB signaling pathway	3	85	0.0100
05222	Small cell lung cancer	3	85	0.0100
05215	Prostate cancer	3	89	0.0100
05200	Pathways in cancer	4	318	0.0476
04062	Chemokine signaling pathway	3	168	0.0476
05214	Glioma	2	63	0.0488
00750	Vitamin B6 metabolism	1	5	0.0488
05211	Renal cell carcinoma	2	70	0.0488
05218	Melanoma	2	71	0.0488
05212	Pancreatic cancer	2	72	0.0488
04810	Regulation of actin cytoskeleton	3	202	0.0488

Table 41: KEGG pathway enrichment for all genes in cluster 2. KEGGID shows the corresponding GO-Term ID. Term indicates the KEGG pathway. Count shows the number of genes found for this pathway and Size is the number of all genes for the corresponding pathway. The p-value is based on the conditional hypergeometric test, corrected by FDR.

KEGGID	Term	Count	Size	*p*-value

Table 42: KEGG pathway enrichment for all genes in cluster 3. KEGGID shows the corresponding GO-Term ID. Term indicates the KEGG pathway. Count shows the number of genes found for this pathway and Size is the number of all genes for the corresponding pathway. The p-value is based on the conditional hypergeometric test, corrected by FDR.

KEGGID	Term	Count	Size	p-value
04930	Type II diabetes mellitus	1	48	0.0180
04920	Adipocytokine signaling pathway	1	66	0.0180
04120	Ubiquitin mediated proteolysis	1	129	0.0180
04630	Jak-STAT signaling pathway	1	134	0.0180
04910	Insulin signaling pathway	1	134	0.0180

Table 43: KEGG pathway enrichment for all genes in cluster 4. KEGGID shows the corresponding GO-Term ID. Term indicates the KEGG pathway. Count shows the number of genes found for this pathway and Size is the number of all genes for the corresponding pathway. The p-value is based on the conditional hypergeometric test, corrected by FDR.

KEGGID	Term	Count	Size	p-value
01100	Metabolic pathways	21	1011	2.4018e-06
00120	Primary bile acid biosynthesis	3	15	0.0021
00830	Retinol metabolism	4	51	0.0033
00980	Metabolism of xenobiotics by cytochrome P450	4	58	0.0042
00072	Synthesis and degradation of ketone bodies	2	9	0.0132
00430	Taurine and hypotaurine metabolism	2	10	0.0137
00982	Drug metabolism - cytochrome P450	3	64	0.0418
04610	Complement and coagulation cascades	3	65	0.0418
03320	PPAR signaling pathway	3	68	0.0422
01040	Biosynthesis of unsaturated fatty acids	2	24	0.0483

Table 44: KEGG pathway enrichment for all genes in cluster 5. KEGGID shows the corresponding GO-Term ID. Term indicates the KEGG pathway. Count shows the number of genes found for this pathway and Size is the number of all genes for the corresponding pathway. The p-value is based on the conditional hypergeometric test, corrected by FDR.

KEGGID	Term	Count	Size	p-value
00982	Drug metabolism - cytochrome P450	9	64	1.7489e-07
01063	Biosynthesis of alkaloids derived from shikimate pathway	6	59	0.0003
01100	Metabolic pathways	22	1011	0.0005
00010	Glycolysis / Gluconeogenesis	5	53	0.0014
04610	Complement and coagulation cascades	5	65	0.0029
00051	Fructose and mannose metabolism	4	37	0.0030
00710	Carbon fixation in photosynthetic organisms	3	21	0.0073
00830	Retinol metabolism	4	51	0.0079
00030	Pentose phosphate pathway	3	26	0.0108
00500	Starch and sucrose metabolism	3	32	0.0179
00120	Primary bile acid biosynthesis	2	15	0.0481

Table 45: KEGG pathway enrichment for all genes in cluster 6. KEGGID shows the corresponding GO-Term ID. Term indicates the KEGG pathway. Count shows the number of genes found for this pathway and Size is the number of all genes for the corresponding pathway. The p-value is based on the conditional hypergeometric test, corrected by FDR.

KEGGID	Term	Count	Size	p-value
04115	p53 signaling pathway	2	63	0.0217
00232	Caffeine metabolism	1	7	0.0434
01100	Metabolic pathways	4	1011	0.0434

Table 46: KEGG pathway enrichment for all genes in cluster 7. KEGGID shows the corresponding GO-Term ID. Term indicates the KEGG pathway. Count shows the number of genes found for this pathway and Size is the number of all genes for the corresponding pathway. The p-value is based on the conditional hypergeometric test, corrected by FDR.

KEGGID	Term	Count	Size	p-value
03030	DNA replication	5	35	0.0073

Table 47: KEGG pathway enrichment for all genes in cluster 8. KEGGID shows the corresponding GO-Term ID. Term indicates the KEGG pathway. Count shows the number of genes found for this pathway and Size is the number of all genes for the corresponding pathway. The p-value is based on the conditional hypergeometric test, corrected by FDR.

FunCluster results

GO-Term enrichment

GOBPID	Term	Count	Size	p-value
GO:0006413	translational initiation	25	14194	2.41e-09
GO:0006412	translation	273	14194	2.36e-49
GO:0006417	regulation of translation	48	14194	1.56e-07

Table 48: GO-Term enrichment for all genes in cluster 1. GOBPID shows the corresponding GO-Term ID. Term indicates the biological process. Count shows the number of genes found for this GO-Term and Size is the number of all genes for the corresponding GO-Term. The p-value is based on the conditional hypergeometric test, corrected by FDR.

GOBPID	Term	Count	Size	p-value
GO:0008380	RNA splicing	169	14194	9.36e-37
GO:0006397	mRNA processing	223	14194	5.12e-40
GO:0016071	mRNA metabolic process	21	14194	1.78e-07
GO:0000398	nuclear mRNA splicing, via spliceosome	22	14194	7.02e-07

Table 49: GO-Term enrichment for all genes in cluster 2. GOBPID shows the corresponding GO-Term ID. Term indicates the biological process. Count shows the number of genes found for this GO-Term and Size is the number of all genes for the corresponding GO-Term. The p-value is based on the conditional hypergeometric test, corrected by FDR.

GOBPID	Term	Count	Size	p-value
GO:0007049	cell cycle	429	14194	3.28e-11
GO:0006915	apoptosis	376	14194	1.99e-17
GO:0051301	cell division	213	14194	4.84e-08
GO:0006260	DNA replication	104	14194	2.09e-06
GO:0051726	regulation of cell cycle	61	14194	2.31e-05

Table 50: GO-Term enrichment for all genes in cluster 3. GOBPID shows the corresponding GO-Term ID. Term indicates the biological process. Count shows the number of genes found for this GO-Term and Size is the number of all genes for the corresponding GO-Term. The p-value is based on the conditional hypergeometric test, corrected by FDR.

GOBPID	Term	Count	Size	p-value
GO:0006511	ubiquitin-dependent protein catabolic process	109	14194	3.01e-11
GO:0006512	ubiquitin cycle	428	14194	2.79e-23

table 62 continued on next page

GOBPID	Term	Count	Size	p-value
GO:0006464	protein modification process	131	14194	6.78e-07

Table 51: GO-Term enrichment for all genes in cluster 4. GOBPID shows the corresponding GO-Term ID. Term indicates the biological process. Count shows the number of genes found for this GO-Term and Size is the number of all genes for the corresponding GO-Term. The p-value is based on the conditional hypergeometric test, corrected by FDR.

GOBPID	Term	Count	Size	p-value
GO:0055114	oxidation reduction	547	14194	2.64e-12
GO:0008152	metabolic process	638	14194	1.19e-15
GO:0006099	tricarboxylic acid cycle	23	14194	4.71e-11
GO:0006629	lipid metabolic process	206	14194	4.82e-06
GO:0008610	lipid biosynthetic process	95	14194	7.79e-06
GO:0008654	phospholipid biosynthetic process	37	14194	1.16e-05

Table 52: GO-Term enrichment for all genes in cluster 5. GOBPID shows the corresponding GO-Term ID. Term indicates the biological process. Count shows the number of genes found for this GO-Term and Size is the number of all genes for the corresponding GO-Term. The p-value is based on the conditional hypergeometric test, corrected by FDR.

GOBPID	Term	Count	Size	p-value
GO:0042254	ribosome biogenesis and assembly	50	14194	1.54e-17
GO:0006364	rRNA processing	60	14194	1.26e-17
GO:0006396	RNA processing	43	14194	1.92e-06

Table 53: GO-Term enrichment for all genes in cluster 6. GOBPID shows the corresponding GO-Term ID. Term indicates the biological process. Count shows the number of genes found for this GO-Term and Size is the number of all genes for the corresponding GO-Term. The p-value is based on the conditional hypergeometric test, corrected by FDR.

GOBPID	Term	Count	Size	p-value
GO:0006810	transport	1612	14194	1.53e-14
GO:0015031	protein transport	453	14194	8.16e-40
GO:0006886	intracellular protein transport	196	14194	7.13e-20
GO:0065002	intracellular protein transport across a membrane	57	14194	1.11e-11
GO:0006754	ATP biosynthetic process	16	14194	1.57e-07
GO:0051028	mRNA transport	43	14194	3.95e-12
GO:0016192	vesicle-mediated transport	152	14194	6.21e-12
GO:0000059	protein import into nucleus, docking	15	14194	4.93e-07

table 54 continued on next page

GOBPID	Term	Count	Size	p-value

Table 54: GO-Term enrichment for all genes in cluster 7. GOBPID shows the corresponding GO-Term ID. Term indicates the biological process. Count shows the number of genes found for this GO-Term and Size is the number of all genes for the corresponding GO-Term. The p-value is based on the conditional hypergeometric test, corrected by FDR.

GOBPID	Term	Count	Size	p-value
GO:0006457	protein folding	114	14194	1.89e-13

Table 55: GO-Term enrichment for all genes in cluster 8. GOBPID shows the corresponding GO-Term ID. Term indicates the biological process. Count shows the number of genes found for this GO-Term and Size is the number of all genes for the corresponding GO-Term. The p-value is based on the conditional hypergeometric test, corrected by FDR.

GOBPID	Term	Count	Size	p-value
GO:0051246	regulation of protein metabolic process	39	14194	8.16e-08
GO:0043687	post-translational protein modification	37	14194	8.01e-08

Table 56: GO-Term enrichment for all genes in cluster 9. GOBPID shows the corresponding GO-Term ID. Term indicates the biological process. Count shows the number of genes found for this GO-Term and Size is the number of all genes for the corresponding GO-Term. The p-value is based on the conditional hypergeometric test, corrected by FDR.

GOBPID	Term	Count	Size	p-value
GO:0045454	cell redox homeostasis	46	14194	3.34e-06

Table 57: GO-Term enrichment for all genes in cluster 10. GOBPID shows the corresponding GO-Term ID. Term indicates the biological process. Count shows the number of genes found for this GO-Term and Size is the number of all genes for the corresponding GO-Term. The p-value is based on the conditional hypergeometric test, corrected by FDR.

GOBPID	Term	Count	Size	p-value
GO:0006986	response to unfolded protein	23	14194	1.69e-05

Table 58: GO-Term enrichment for all genes in cluster 11. GOBPID shows the corresponding GO-Term ID. Term indicates the biological process. Count shows the number of genes found for this GO-Term and Size is the number of all genes for the corresponding GO-Term. The p-value is based on the conditional hypergeometric test, corrected by FDR.

Pathway enrichment

KEGGID	Term	Count	Size	p-value
00020	Citrate cycle (TCA cycle)	27	4165	5.73e-08
00190	Oxidative phosphorylation	128	4165	3.16e-12

Table 59: KEGG pathway enrichment for all genes in cluster 1. KEGGID shows the corresponding GO-Term ID. Term indicates the KEGG pathway. Count shows the number of genes found for this pathway and Size is the number of all genes for the corresponding pathway. The p-value is based on the conditional hypergeometric test, corrected by FDR.

KEGGID	Term	Count	Size	p-value
04110	Cell cycle	109	4165	7.44e-06
04120	Ubiquitin mediated proteolysis	132	4165	3.80e-09
03420	Nucleotide excision repair	41	4165	2.02e-05

Table 60: KEGG pathway enrichment for all genes in cluster 2. KEGGID shows the corresponding GO-Term ID. Term indicates the KEGG pathway. Count shows the number of genes found for this pathway and Size is the number of all genes for the corresponding pathway. The p-value is based on the conditional hypergeometric test, corrected by FDR.

KEGGID	Term	Count	Size	p-value
03050	Proteasome	31	4165	4.21e-12

Table 61: KEGG pathway enrichment for all genes in cluster 3. KEGGID shows the corresponding GO-Term ID. Term indicates the KEGG pathway. Count shows the number of genes found for this pathway and Size is the number of all genes for the corresponding pathway. The p-value is based on the conditional hypergeometric test, corrected by FDR.

KEGGID	Term	Count	Size	p-value
03010	Ribosome	93	4165	3.05e-10

Table 62: KEGG pathway enrichment for all genes in cluster 4. KEGGID shows the corresponding GO-Term ID. Term indicates the KEGG pathway. Count shows the number of genes found for this pathway and Size is the number of all genes for the corresponding pathway. The p-value is based on the conditional hypergeometric test, corrected by FDR.

KEGGID	Term	Count	Size	p-value
00071	Fatty acid metabolism	43	4165	8.2e-05
00280	Valine, leucine and isoleucine degradation	43	4165	5.48e-06

Table 63: KEGG pathway enrichment for all genes in cluster 5. KEGGID shows the corresponding GO-Term ID. Term indicates the KEGG pathway. Count shows the number of genes found for this pathway and Size is the number of all genes for the corresponding pathway. The p-value is based on the conditional hypergeometric test, corrected by FDR.

KEGGID	Term	Count	Size	p-value
04610	Complement and coagulation cascades	71	4165	8.71e-05

Table 64: KEGG pathway enrichment for all genes in cluster 6. KEGGID shows the corresponding GO-Term ID. Term indicates the KEGG pathway. Count shows the number of genes found for this pathway and Size is the number of all genes for the corresponding pathway. The p-value is based on the conditional hypergeometric test, corrected by FDR.

Clustering of significantly regulated genes

All Affymetrix GeneChip arrays were preprocessed as described in the Methods section. To find significantly expressed genes, for every available gene, i.e. probe set, a t-test was applied to compare the expression level of time point t with the reference time 0h. In order to cope with the occurring multiple-testing problem for 45101 probe sets, the obtained p-values are corrected with the false-discovery- rate method. Finally we obtained a set of 121 genes having a p-value ≤ 0.05 and a log 2 change of 1. Moreover, we applied a k-means clustering to temporally group this set into 4 cluster. The following tables show the corresponding gene for each cluster

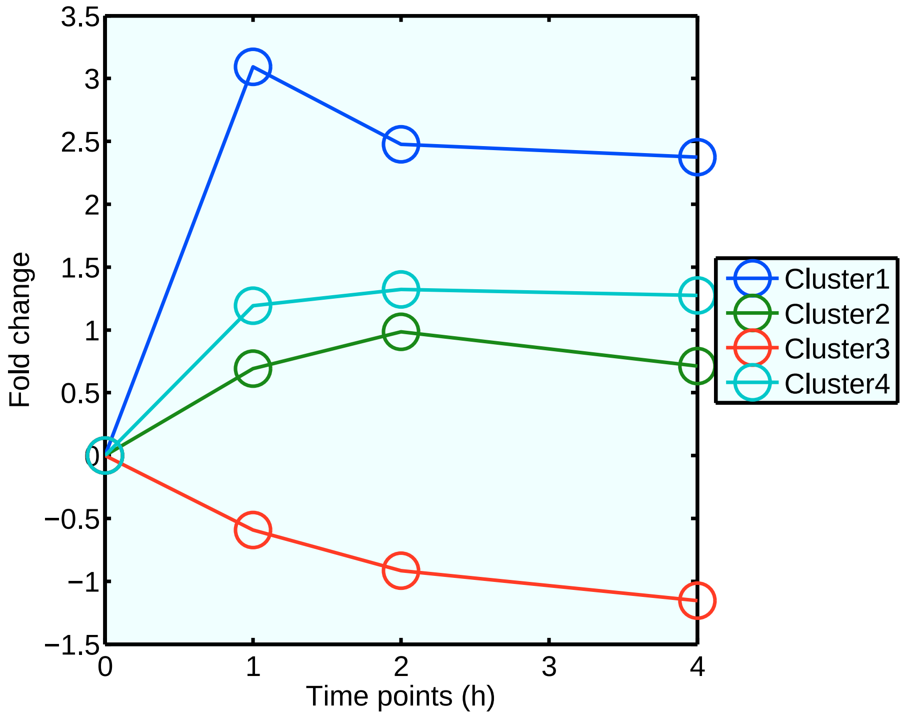

Additional Figure 2: The result of the *k*-means clustering is plotted. The x-axis shows the measured time-points and the y-axis shows the fold-change values of the centroids at that time-points.

symbol	h0	h1	h2	h4	clusterID
Socs3	0	2.50466539620936	1.89227520948198	1.85314671281972	1
Cebpd	0	3.03024565759234	2.70106317171277	2.47159033489943	1
Socs3	0	3.56839350658948	2.81312781829989	2.64083521467781	1
Socs3	0	3.26535756654752	2.50154542100266	2.53143788831907	1

Table 65: Significantly expressed genes in Cluster 1. h0 to h4 show the foldchange expression values of the time points h0 to h4. clusterID indicates the *k*-means clustering result.

symbol	h0	h1	h2	h4	clusterID
Lox	0	0.599688943018913	1.13180927818671	0.61355677349155	2
Rgs4	0	0.779733603493623	0.960341748113397	1.09377295224598	2
Shcbp1	0	0.565935789254327	1.00920909886051	0.68046278657621	2
Nusap1	0	0.604878047128754	1.10359621653723	0.891836435490574	2
Krt19	0	0.197564799154193	1.08432092136183	0.82941780852341	2
Pdk4	0	1.15637836086362	0.47136263396362	-0.268104984457183	2
Tacc3	0	0.557726233911249	1.11407079821826	0.916790840209357	2
Bcl3	0	0.728421529181743	1.03486227826536	1.05930973703049	2
Cenpk	0	0.363630369521497	1.07160320954147	0.462658216630013	2
Iigp1	0	0.879507913909888	0.984757433804456	1.05117910547649	2

Table 66 continued on next page

symbol	h0	h1	h2	h4	clusterID
Fgfbp1	0	0.381727800628916	1.1308406954528	0.159936827055366	2
Pde4b	0	1.32501632358617	0.26128225444729	0.296876127989711	2
Serpinb9b	0	0.240339003762871	1.27379244424626	0.54257004292072	2
Diap3	0	0.398591024435987	1.00510781018977	0.586867828554123	2
Bub1	0	0.746150937222707	1.20984016425229	0.910581156778624	2
Aurkb	0	0.617892836890586	1.07814780510738	0.774401238856367	2
Birc5	0	0.528212983441056	1.07016542182948	0.74894733444736	2
Trim6	0	1.53022201044067	0.35782861052949	0.4009161373787	2
Tspan8	0	0.66911457566478	1.29659384543018	0.98279039732244	2
Hmmr	0	0.59134477774368	1.01174501889515	0.607208366467017	2
Steap4	0	0.724937496373279	0.77609072951561	1.09209293317711	2
Id1	0	0.304899764484486	1.47756580221389	0.754554975774946	2
Glul	0	0.88724016083109	0.706216374477758	1.03805962987822	2
Mki67	0	0.55450755963028	1.04124357975626	0.826802435721357	2
Cenpf	0	0.52813085762227	1.01963333013095	0.57765743507279	2
4833442J19Rik	0	0.537648182831383	0.940922884750156	1.21763904073789	2
A630038E17Rik	0	0.414911472447036	1.41317533929435	0.75845782773805	2
Tpx2	0	0.65689181420802	1.03864487329355	0.722977045737213	2
Ncapg	0	0.51506166831752	1.13909568667022	0.85950619198556	2
Hfe2	0	1.01612226610016	1.01624865773551	0.58815746717849	2
Bmper	0	0.345287936361797	1.18027658877259	1.40308131307235	2
Oip5	0	0.508143077931547	1.00358583124711	0.559285899130353	2
Nuf2	0	0.783035857904897	1.27441369882328	0.813483391563747	2
Csrnp1	0	1.06525622387732	0.492779825742836	0.167764435772622	2
C730029A08Rik	0	0.36720087501152	0.678177320643957	1.14405597133941	2
BC046404	0	0.321320611681037	1.10292731492934	0.732518872350007	2
Sgms1	0	1.11083953637192	0.88793255213679	0.473985899774487	2
D630045M09Rik	0	0.588756714307763	1.05573152316052	0.708660243656636	2
2010109K11Rik	0	1.02669823146272	0.880508816395206	0.730583370723137	2
Pim3	0	1.266675161236	0.780937616047211	0.78485282633243	2
Foxq1	0	1.23011303320323	0.67245852381089	0.530095465186794	2
Lox	0	0.599734998900523	1.08404941715175	0.599828089297203	2
Cish	0	1.00451061081399	0.420702415015147	0.566782272163923	2
Il1r1	0	0.483970596841517	0.92745226622691	1.06657893204159	2
Ttk	0	0.660021401422811	1.09609294211319	0.708349576142208	2
Saa2	0	0.558120848080553	1.2170354402785	0.467976246407473	2
Nrk	0	0.385022062477767	1.0104316217658	0.745264798177293	2
Nrk	0	0.484332359151948	1.12586126883087	0.847746010251984	2
Hmmr	0	0.722371871059367	1.04535084103646	0.57054042237142	2
Saa1	0	0.558585936708399	1.09076856493837	0.527607496475833	2
Ccnb2	0	0.731481442543867	1.01455720714019	0.743016382569394	2
Klf5	0	0.520498410238303	1.17514524766829	0.526621025603246	2
Pim3	0	1.24147474036018	0.705752040495427	0.658730124949694	2
Cdca3	0	0.731930310861804	1.05484085511371	0.7310368489887	2
Cep55	0	0.626164138374273	1.07123752868014	0.732039453263184	2
Dscc1	0	0.602355644534653	1.09359420871517	0.223768654044817	2
Gpr146	0	1.21899481315972	0.897649335795354	0.56958292758363	2

Table 66 continued on next page

symbol	h0	h1	h2	h4	clusterID
Steap4	0	0.8129278336626	0.833194928124954	1.15291123871684	2

Table 66: Significantly expressed genes in Cluster 2. h0 to h4 show the foldchange expression values of the time points h0 to h4. clusterID indicates the k-means clustering result.

symbol	h0	h1	h2	h4	clusterID
Il33	0	1.14815490023132	1.45985020353923	1.45966834964575	3
Efna1	0	1.24055015418258	0.877810280550757	1.1780546788567	3
Timd2	0	0.912478394079093	1.64920684505007	1.40616486521867	3
Hamp	0	1.14591727170784	2.2071483165769	1.54491272370206	3
Hamp	0	1.05682802014865	2.13760783122603	1.48434055976459	3
Gcnt2	0	1.18845392714787	0.965872817097807	0.99969967767617	3
Racgap1	0	0.828003666566937	1.25898340603929	1.11374517556923	3
Ahr	0	1.00393791971309	1.1886437594587	1.09529745379281	3
Gcnt2	0	1.20934542593594	0.959218199424543	1.01070984615311	3
Tifa	0	1.79130034390001	1.79366692727092	1.78535400525412	3
Arrdc4	0	1.64692632479319	1.24224984508847	1.48199369216042	3
Gcnt2	0	1.22298991426492	1.02408590994308	1.23867519783555	3
Ypel2	0	1.25269821364789	1.29079636861	0.963459006595946	3
Cp	0	1.06041697816563	0.80099807249105	1.16044020712491	3
Efna1	0	1.25823730939402	0.87363700711638	1.22108920043289	3
Tgm1	0	0.97147473901921	1.12253505622533	1.47621464571827	3
Gcnt2	0	1.2476127266439	1.0650301414775	1.06783034997381	3
Mbd1	0	1.17804315556752	1.87671797780932	1.56403055299249	3
Ypel2	0	1.30950589365279	1.32478844702548	0.972298753469283	3

Table 67: Significantly expressed genes in Cluster 3. h0 to h4 show the foldchange expression values of the time points h0 to h4. clusterID indicates the k-means clustering result.

symbol	h0	h1	h2	h4	clusterID
Per2	0	-1.04331500135682	-1.64215128176287	-0.872444381780307	4
Vldlr	0	-0.81302827534408	-1.14555434114857	-0.945669695657946	4
Egln3	0	-1.76683631419645	-1.8652479046968	-2.419386622903	4
Sgk2	0	-0.476051320919376	-0.723792287196806	-1.12727401968676	4
Tdo2	0	0.0353543487171546	-0.515734757504479	-1.27360155814155	4
Serpine1	0	-0.98262376918705	-1.34972796656261	-1.21072635937021	4
Defb1	0	-0.529306539137747	-0.967437798430903	-1.51203807453687	4
Arg1	0	0.124277997243293	-0.541609219872431	-1.52659038955554	4
Hpgd	0	-0.741299493994837	-1.04387937121482	-1.61413979435464	4
Il17rb	0	-0.435122212644133	-0.896765347381657	-1.12237167145281	4
Cyp1a1	0	-0.7523657162294	-1.29857140650239	-1.48156433727134	4

Table 68 continued on next page

symbol	h0	h1	h2	h4	clusterID
Uox	0	-0.740823562464879	-1.19997783819858	-1.60561296251426	4
Fgf21	0	-0.98754572558394	-0.753924108788233	-1.03567626463216	4
Cldn23	0	-0.487537253403377	-1.10573685392978	-1.02143669761633	4
Acaa1b	0	-0.727411911064077	-1.1495121420066	-1.04175941191582	4
Angptl3	0	-0.509657983397908	-1.09297818266208	-1.14082895691391	4
Lmcd1	0	-0.836116951983727	-1.42414350811894	-1.02413023998399	4
Acnat2	0	0.125966781293016	-0.477659862312207	-1.10555257551379	4
Rorc	0	-0.20649108213309	-0.61626297556919	-1.0260275364576	4
Zfp655	0	-0.24981868613825	-0.768754937457437	-1.04482712549064	4
Pdlim5	0	-0.511494772642614	-0.539440212066298	-1.07844442825177	4
Rhpn2	0	-0.42354312160227	-0.69958701683384	-1.04900319082605	4
Vldlr	0	-0.75820751502184	-0.88815013470959	-1.01716588661571	4
Rhpn2	0	-0.373755355551006	-0.687253850378864	-1.0361267336552	4
Ces3	0	-0.674557508995527	-0.677115812211373	-1.13594283742107	4
Ankrd37	0	-1.26252989556952	-1.13956620439447	-1.15718028973681	4
Cldn1	0	-0.186750213472227	-0.726248719726127	-1.19896496456459	4
Dbp	0	-0.91312371148094	-1.33425029417551	-0.130860193635493	4
Gpr120	0	-0.927879158131204	-1.10053263823768	-1.01857391042685	4
Zmiz1	0	-1.10742520872932	-0.450298133322174	0.011880293356973	4
G0s2	0	-1.17281315358806	-1.15211773890441	-0.894435379463347	4
Tdo2	0	-0.12530722782825	-0.73096545955135	-1.45014074281632	4
Cldn1	0	-0.213284196744606	-0.619956742206803	-1.21216106904948	4
Cyp1a2	0	-0.728377316160537	-0.861384278906247	-1.19780648370025	4
Slc26a1	0	-0.284020057756226	-0.75424012836245	-1.17646456554638	4
Srxn1	0	-0.288140682946436	-0.422370544919163	-1.05601777478659	4
1600029D21Rik	0	-0.388511626424776	-0.687628099184273	-1.10557086647981	4
Chdh	0	-0.318083741570454	-0.95823642018068	-1.46736909809099	4
Slc26a1	0	-0.388785846837723	-0.788007422164614	-1.22943957488909	4
Thbs1	0	-0.650372577963546	-0.827680545078033	-1.41257877119732	4

Table 68: Significantly expressed genes in Cluster 4. h0 to h4 show the foldchange expression values of the time points h0 to h4. clusterID indicates the k-means clustering result.

Yeast cell cycle data

The yeast cell cycle data were published by Cho et al. (1998). The data contains the expression profiles of 6220 genes over 17 time points taken at 10-min intervals. This set of data has been analyzed by various studies, e.g. Tamayo et al. (1999), Lukashin and Fuchs (2001), Yeung et al. (2001), Liao (2003) and Qu (2004) . The entire dataset is available at `http://genome-www.stanford.edu/cellcycle/`. The gene regulatory network used as prior knowledge for GraDe was obtained from `http://web.wi.mit.edu/young/regulator_network/`. We used the data set and applied GraDe, PCA, k-means clustering, and the FunCluster method on the data.

We evaluated the four different methods by taking the five cell division phases (early G1, late G1, S, G2, and M) into account. We assigned for each method and cell cycle phase the best source or cluster containing the highest amount of genes of the particular cell cycle phase. Table 69 summarizes the results obtained for the four different methods. Additional Figure 3 shows the time-course pattern of the resulting sources or clusters for GraDe, PCA, k-means clustering, and FunCluster.

Cell cycle phase	GraDe	PCA	k-means	FunCluster
early G1	$S7_{neg}$ (23 (21%))	$S2_{pos}$ (27 (23%))	$C16$ (19 (63%))	
late G1	$S2_{neg}$ (58 (41%))	$S3_{pos}$ (49 (36%))	$C8$ (47 (18%))	$C11$ (54 (23%))
S	$S2_{neg}$ (10 (7%))	$S2_{pos}$ (11 (11%))	$C8$ (14 (5%))	$C11$ (54 (23%))
G2	$S9_{neg}$ (8 (8%))	$S1_{pos}$ (6 (4%))	$C12$ (11 (2%))	
M	$S2_{pos}$ (16 (23%))	$S1_{pos}$ (14 (10%))	$C17$ (16 (40%))	$C12$ (131 (54%))

Table 69: The table summarizes the results for the four different methods. S and C indicate source and cluster, respectively. Pos and Neg indicate a positive or negative source contribution. The numbers in brackets are the numbers of genes found for the particular cell cycle phase. The percentage indicates the fraction of cell cycle genes compared to all gene in the particular source or cluster.

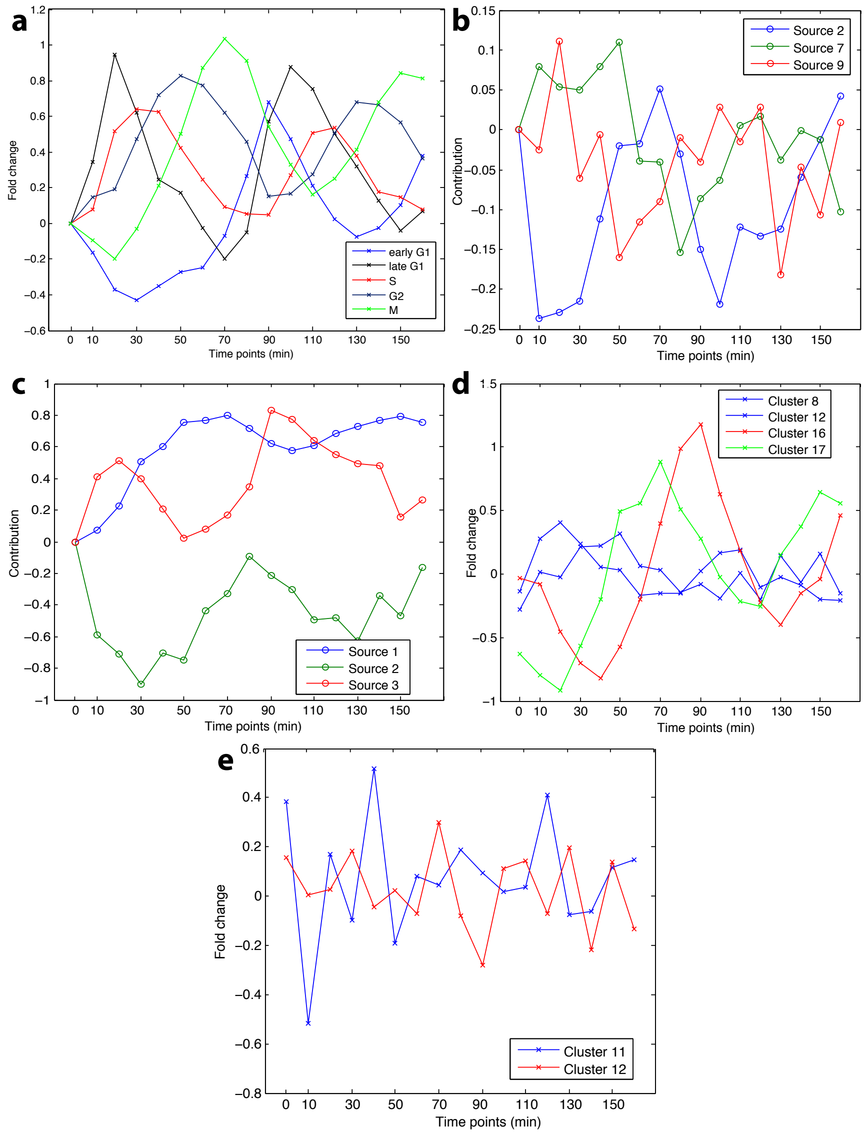

Additional Figure 3: Time-course profiles. (**a**) shows the time-course pattern for the five different cell division phases. (**b-e**) show the time-course expression profiles for GraDe, PCA, k-means, and FunCluster. The x-axis shows the 17 different data points. The expression profiles were measured for 160 minutes at 10-min intervals. Source contribution or fold change values are illustrated by the y-axis.

The yeast cell cycle genes had expression levels peaking at different times corresponding to the five phases of the cell cycle (see Additional Figure 3a). We used these five different sets of genes to assign the best source or cluster for each method at a particular cell cycle phase. The time-course patterns of GraDe correctly identified the expression profiles of the cell cycle genes in the early G1 phase. We found the highest amount of genes for the early G1 phase in the source 7 with genes having a negative contribution. In total, we identify 23 early G1 genes (compare Table 69). The time-course expression pattern of genes in source 7 having a negative contribution shows the characteristic decrease in expression until 30-40 minutes and the increase in expression with the highest peak at 80 minutes (see Additional Figure 3b). Moreover, GraDe also identified the following decrease with an increasing pattern at 160 minutes. Comparing the result of PCA, we found the highest amount of genes for the early G1 phase in Source 2. This source also correctly showed the decrease until 30 minutes followed by the activation peak at 80 minutes (see Additional Figure 3c). K-means identify a reasonable amount of early G1 genes but cluster 16 failed to find the correct time-course profile (see Additional Figure 3d). Using the FunCluster method, which incorporates gene annotation and gene expression into the clustering task, we did not find a specific cluster assigned to the early G1 phase.

The late G1 phase shows an early activation peak at 20 minutes followed by a decreasing until 100 minutes. A second peak was identified at 110 minutes (see Additional Figure 3a). Using GraDe, we found these genes grouped in source 2 having a negative contribution. Again, this source perfectly identified the exact time pattern of the late G1 phase with both activation peaks and decreasing phases. For the PCA we identify the highest amount of late G1 genes in Source 3. This source also founds both activation peaks at 20 and 100 minutes. Compared to GraDe, PCA identified less late G1 genes and grouping more genes in the particular source. K-means obtain a similar result to PCA in the amount of identifies genes, however the resulting cluster contained only 18% of late G1 genes. Moreover, the time-course of cluster 8 shows only the first activation peak at 20 minutes. FunCluster grouped the late G1 phase by identifying a similar amount of genes compared to GraDe. However, cluster 11 showed none of the characteristic activation peaks obtained by GraDe or PCA (see Additional Figure 3e).

Genes of the S phase show a similar expression pattern to the late G1 phase. The first activation peak is shifted by 10 minutes followed by the same phase of decreasing expression profiles. The second peak is then again shifted by 10 minutes compared to the late G1 phase (see Additional Figure 3a). GraDe and k-means identified S phase related genes in the same source or cluster as the late G1 genes, reflecting the very similar time-course expression pattern. But again, only the time-course profile of GraDe reflected the activation peaks and decreasing phases obtained by the time-course data set. Different to GraDe and k-means, PCA identified S phase genes in the same source as the early G1 genes. Hence, the time-course profile did not reflect the correct expression profile for the S-phase genes. FunCluster identified S-phase genes in the same cluster as the late G1 genes, similar to GraDe and k-means. Based on a combined annotation of late G1 and S phase, FunCluster identified the highest amount of S phase genes compared to the other methods.

The G2 phase genes showed a strong increase in expression after 50 minutes followed by a decreasing until 90 minutes and a second peak at 130 minutes. GraDe grouped these genes in Source 9 having a negative contribution. Again, GraDe was able to detect the first and second activation peak at 50 and 130 minutes, respectively. However, GraDe identified only 8 genes of the G2 phase in this source. A similar result was obtained for PCA identifying only 6 genes in source 1. Moreover, Source 1 showed the activation peak at 70 minutes but failed to find the decreasing phase and second activation peak. K-means identified the highest number of G2 genes but cluster 12 failed to find the time-course expression profile of G2 genes in a proper way. Using the FunCluster method, we did not find a specific cluster assigned to the G2 phase.

The final M phase starts with a decreasing phase until 20 minutes followed by an activation peak at 70 minutes and repeating pattern until 160 minutes. GraDe correctly detected this time-course pattern using Source 2. PCA identified a similar amount of M phase genes compared to GraDe but grouped these genes into Source 1, which failed to find the time-course expression pattern. K-means perfectly identified the time-course expression profile of the M phase. FunCluster found the highest

amount of M-phase genes in cluster 12. However, the cluster 12 failed to find the expression profile of the M phase.

In comparison to PCA, k-means and FunCluster, GraDe was the only method, which correctly identified all five different yeast cell cycle expression profiles. In addition to the analyzed data set of *IL-6* stimulated primary mouse hepatocytes, we were able to show that including prior knowledge into the separation task leads to a much more structured and detailed separation of the time-dependent responses compared to standard techniques such as PCA, k-means or FunCluster.

Illustration of GraDe

Bifan topology

As illustrated in Figure 3a, we used for the first toy example a bifan structure. We assume to have six nodes from a stimulated time-course. The system is described by ordinary differential equations and interactions are modeled by sigmoidal hill functions. We set the hill coefficient for all reactions to 3. The dissociation was set to 1 and the ligand concentration to 0.00001 for all reactions.

A Matlab file containing the expression values shown in Figure 3b is available on our webpage `http://cmb.helmholtz-muenchen.de/grade/`. This file contains also the network matrix illustrated in Figure 3a.

Funnel topology

We generated a funnel structure for the second toy example. For this example, we generated node expressions for three different conditions. The node expression for condition 1-3 is shown in the Additional Table 70.

A Matlab file containing the expression values shown in Table 70 is available on our webpage `http://cmb.helmholtz-muenchen.de/grade/`. This file contains also the network matrix illustrated in Figure 3f.

Node	Condition 1	Condition 2	Condition 3
1	0	1	1
2	1	0	1
3	0	1	.5
4	0	1	.5

Table 70: Node expression during the three different conditions based on our second toy example. 1 indicates an active node, 0 an inactive.

Examples of overlapping clusters

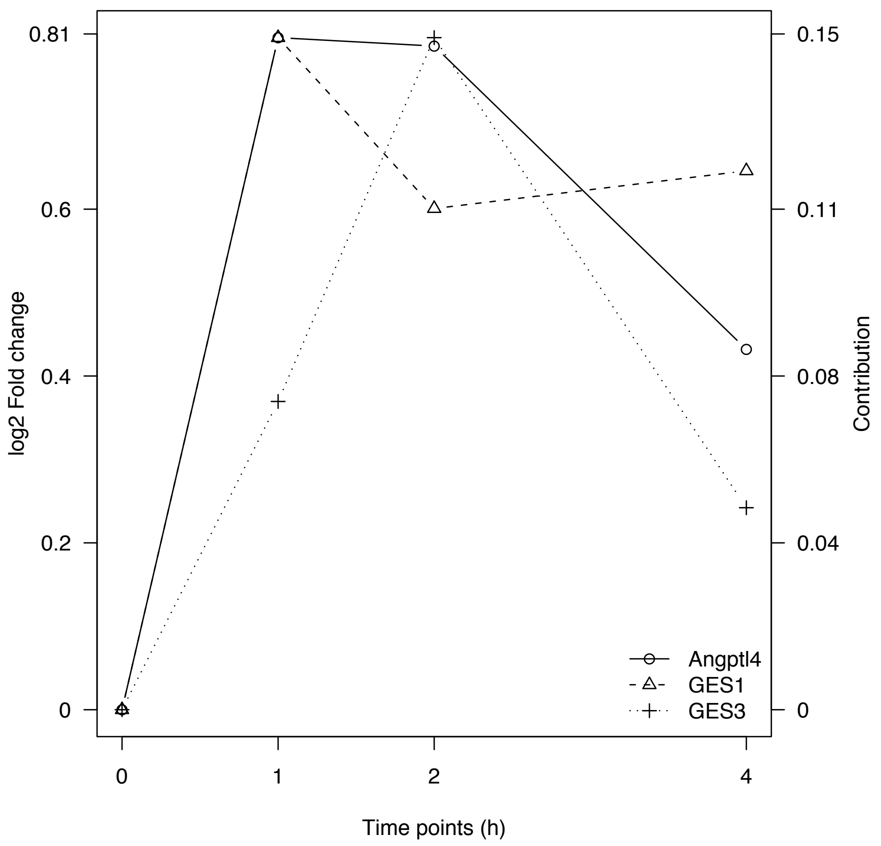

Additional Figure 4: *Angptl4* is a positive acute phase protein showing a strong increase in expression during the first hour after stimulation followed by a decrease after two hours (see Additional Material). We identify *Angptl4* in GES 1 and 3 showing perfectly the strong increase after IL-6 (GES 1) and the induced decreased after 2 hours (GES 3).

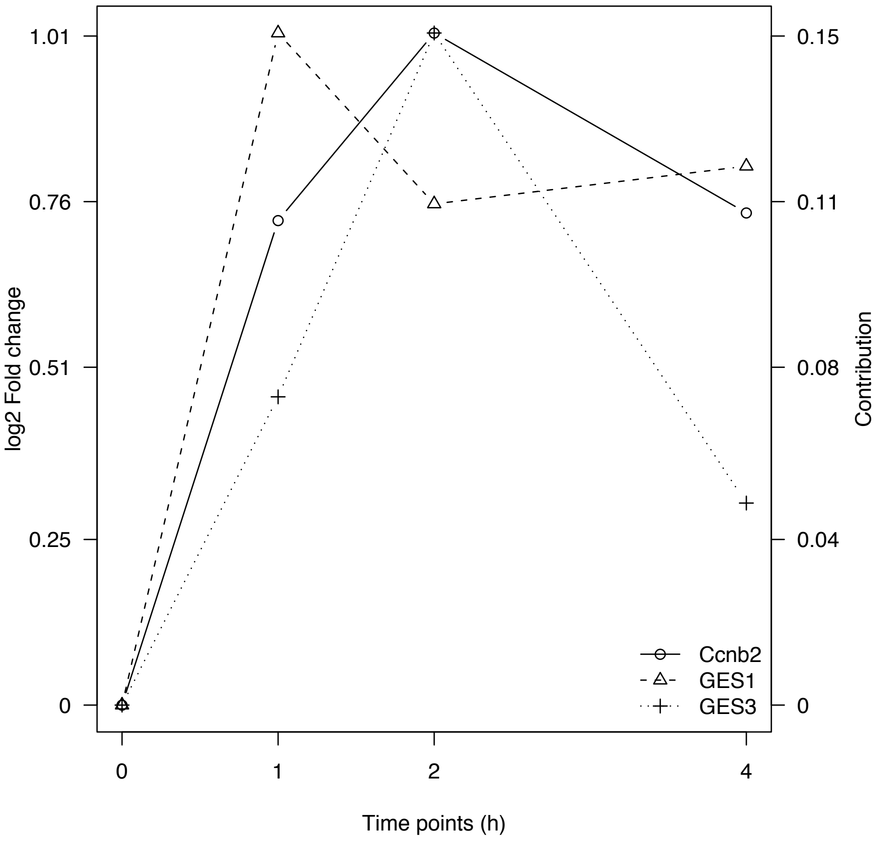

Additional Figure 5: *Ccnb2* a late cell cycle genes, which repression leads to cell cycle arrest in the G2 phase. The time-course expression pattern, shows a strong increase after *IL-6* stimulation followed by a decrease after two hours. We identify *Ccnb2* in GES 1 and GES 3 perfectly reconstruct the strong increase after the stimulation and the inactivation after two hours.
